# Multinuclear Zinc–Magnesium Hydroxide Carboxylates: A Predesigned Model System for Copolymerization of CO_2_ with Epoxides

**DOI:** 10.1021/acs.inorgchem.3c02177

**Published:** 2023-09-15

**Authors:** Vijay Gupta, Iwona Justyniak, Elżbieta Chwojnowska, Vadim Szejko, Janusz Lewiński

**Affiliations:** † Faculty of Chemistry, Warsaw University of Technology, 00-664 Warsaw, Poland; ‡ Institute of Physical Chemistry, Polish Academy of Sciences, 01-224 Warsaw, Poland

## Abstract

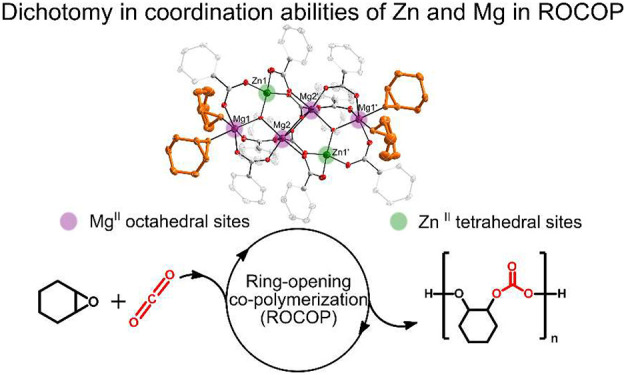

Among numerous catalysts in the ring-opening copolymerization of epoxides with carbon dioxide (CO_2_), zinc dicarboxylate complexes are the most common type, and in the family of metal-based homogeneous catalysts, zinc and magnesium complexes have attracted widespread attention. We report on the synthesis and structural characterization of a zinc–magnesium benzoate framework templated by the central hydroxide anion with μ_3_-κ^2^:κ^2^:κ^2^ coordination mode, [ZnMg_2_(μ_3_-OH)(O_2_CPh)_5_]_*n*_ (*n* = 1 or 2). The resulting heterometallic system forms stable Lewis acid–base adducts with tetrahydrofuran (THF) and cyclohexene oxide (CHO), which crystallize as the hexanuclear zinc–magnesium hydroxide carboxylate cluster [ZnMg_2_(μ_3_-OH)(O_2_CPh)_5_(L)_2_]_2_ (L = THF or CHO). Their X-ray crystal structure analysis revealed that the Zn center prefers 4-fold coordination and the Mg centers demonstrated the ability to accommodate higher coordination numbers, and as a result, the heterocyclic molecules are exclusively bonded to 6-fold Mg atoms. The heteronuclear carboxylate aggregates appeared active in the copolymerization reaction at elevated temperatures to produce an alternating poly(cyclohexene carbonate).

## Abstract 2

A novel multinuclear heterometallic model system for ring-opening copolymerization (ROCOP) of epoxide/carbon dioxide is reported.

The ring-opening polymerization (ROP) of epoxides^1^ and copolymerization (ROCOP) of epoxides [e.g., propylene oxide (PO) and cyclohexene oxide (CHO)] with carbon dioxide (CO_2_),^1d,2^ leading to polyethers and polycarbonates, respectively, have received significant attention over the past decades due to biocompatibility, biodegradability, and widespread applications of the resulting materials. In 1969, Inoue and co-workers discovered a heterogeneous catalyst for the alternating copolymerization of PO and CO_2_ employing a ZnEt_2_/H_2_O reaction system,^3^ and this pioneering work has continued to captivate scientific attention and hence resulted in the development of many metal-based catalysts to produce alternating polycarbonates,^2a,4^ including catalytic systems for the enantioselective copolymerization of cycloalkene oxides and CO_2_.^5^ Among the plethora of homogeneous catalytic systems, zinc complexes have attracted widespread attention,^5a,5b,6^ and, in particular, a bimetallic zinc catalyst reported by Coates and co-workers led to the development of the bimetallic family of catalysts.^4b,4c,6b−6d,6f^ The dinuclear systems with two metal centers in close proximity usually exhibited enhanced catalytic activity in the ROCOP due to the cooperative effect. The synergic interaction mechanism in homobimetallic catalysts has motivated scientists to develop catalytic systems featuring numerous heterometallic combinations from across the s, p, and d/f-block elements.^7^ Notably, zinc(II)–magnesium(II)^8^ and cobalt(II)–magnesium(II)^9^ heterodinuclear systems with macrocyclic ligands are the most active and selective. The catalytic pathways of the ROCOP are considered to be a chain polymerization involving a coordination–insertion mechanism,^10^ and in the case of zinc–magnesium heterodinuclear systems, their enhanced activity has been interpreted as a result of the chain shuttling mechanism, with Lewis acidic magnesium enhancing epoxide coordination and the labile zinc carbonate bond accelerating the nucleophilic attack.^8^ While a vast number of various types of structurally well-defined mononuclear and di/multinuclear catalysts for the ROCOP have been developed,^4−9^ both the respective Lewis acid–base adduct with heterocyclic monomers and the intermediate formed by the monomer primary insertion event are very scant.^11^ In turn, many metal complexes can insert CO_2_ molecules into M–H, M–C, M–N, M–OH, and M–OR bonds to afford M–OC(O)OX.^12^

Meanwhile, heterogeneous catalysts such as double-metal cyanide and zinc dicarboxylate complexes^13^ are also known and present their own attractiveness with regard to an industrial point of view.^14^ However, the exact structure of these heterogeneous catalysts has yet to be clarified, and as noted by Chisholm and co-workers, the challenge thus remains to design a molecular system that mimics the active site.^17a^ Among various heterogeneous dicarboxylate-based zinc initiators/catalysts, zinc glutarates seem to be particularly promising in producing an alternating copolymer in high yield,^15^ albeit innumerable consecutive attempts could not lead to the desired high activities^16^ that have been realized for several homogeneous catalysts.

As a part of our study on the activation of heterocyclic monomers by the metal complexes,^18^ keeping in mind both the highly effective homogeneous zinc–magnesium heteronuclear systems^8^ and the lack of a well-defined molecular system for heterogeneous zinc carboxylate-based catalysts (interestingly, some authors have inferred that likely Zn–OH sites are highly active in producing long-chain polymers^17^), herein we report on the first structurally characterized multinuclear zinc–magnesium hydroxide cluster supported by a simple carboxylate ligand. The resulting heterometallic system forms stable Lewis acid–base adducts with CHO, which represents an isolated intermediate in the initiation of ROCOP catalyzed by a heterometallic zinc–magnesium system. Moreover, the heteronuclear carboxylate aggregates appeared to be active in the ROCOP of CHO/CO_2_ at elevated temperatures to produce an alternating poly(cyclohexene carbonate).

In the course of our ongoing effort to develop effective synthetic methods of various metal carboxylate aggregates,^19^ we previously designed and developed a two-step reaction system for the atom-efficient preparation of [Zn_4_(μ_4_-O)(O_2_CR)_6_]-type clusters^19a^ as a predesigned platform for modeling the reactivity of prototypical Zn-based metal-organic framework (MOF-5) toward water (H_2_O) and donor solvents.^18d^ The synthetic procedure involved the reaction of diethylzinc (Et_2_Zn; 2 equiv) with a carboxylic acid (3 equiv) in a tetrahydrofuran (THF) solution followed by the addition of H_2_O (0.5 equiv). Now we extended this procedure for the preparation of soluble heteronuclear zinc–magnesium oxide/hydroxide carboxylates. Initially, in a control experiment, ZnEt_2_ (2 equiv) and ^n^Bu_2_Mg (2 equiv) were reacted with benzoic acid (6 equiv) in a THF solution in the temperature range of −78 to +20 °C, and then 1 equiv of H_2_O was added (for details, see the Supporting Information). The reaction afforded a complex mixture of products. After a standard workup and crystallization, a hexanuclear zinc–magnesium carboxylate framework templated by a central hydroxide anion with a μ_3_-κ^2^:κ^2^:κ^2^-coordination mode, [Zn_2_Mg_4_(μ_3_-OH)_2_(O_2_CPh)_10_(THF)_4_] (**1-THF**), along with a linear trinuclear magnesium carboxylate, [Mg_3_(O_2_CPh)_6_(THF)_4_] (**2**), was obtained. Next, **1-THF** was prepared in essentially quantitative yield based on the atom-economy approach, i.e., in a one-pot stoichiometric reaction of Et_2_Zn (1 equiv) and ^n^Bu_2_Mg (2 equiv) with benzoic acid (5 equiv) in THF, followed by the addition of H_2_O (1 equiv; Scheme 1). **1-THF** was characterized spectroscopically, and its molecular structure was confirmed by single-crystal X-ray crystallography.

**Scheme 1:**
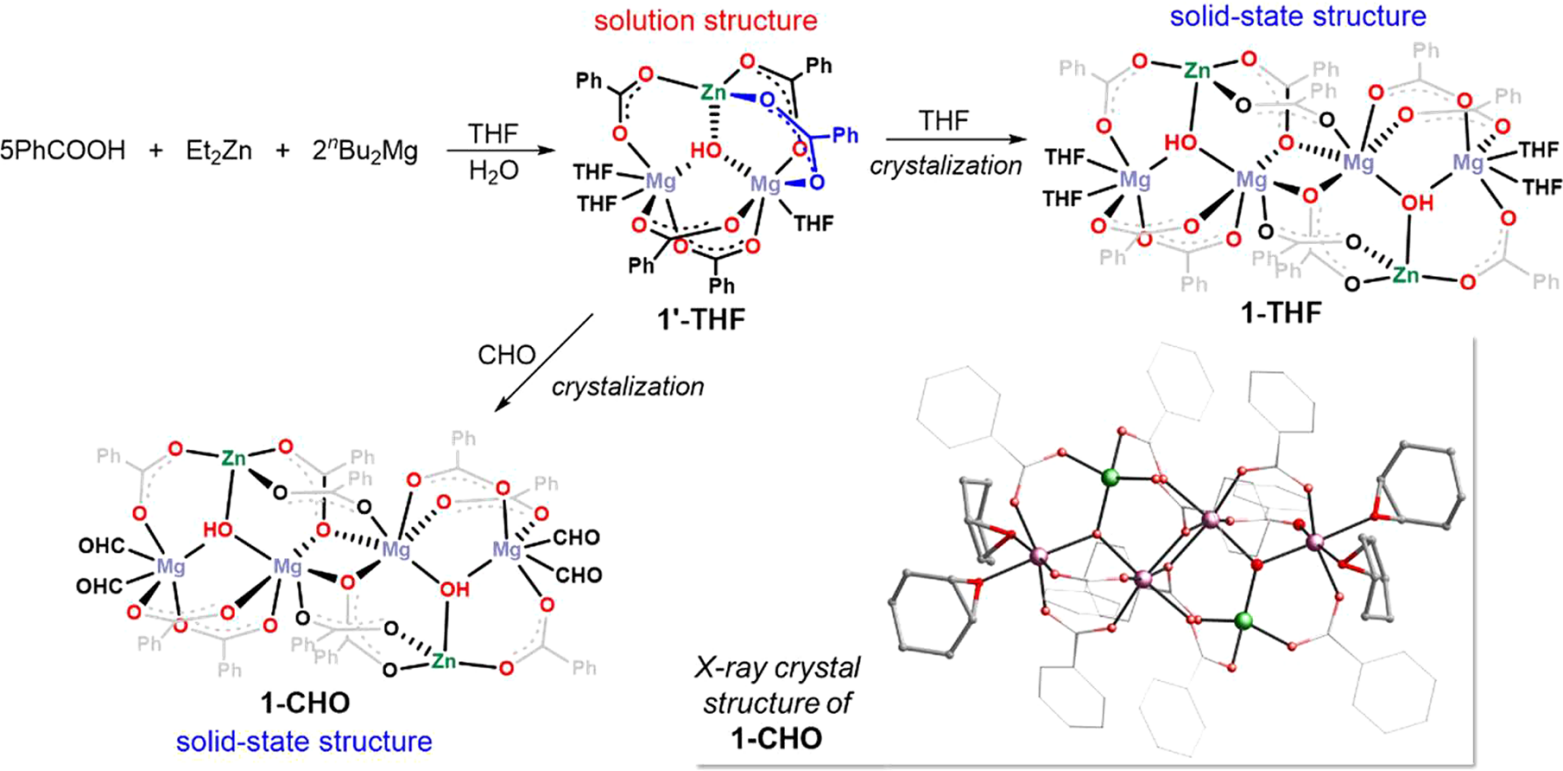
Synthesis of Hexanuclear Zinc–Magnesium Hydroxide Carboxylate **1-THF** and the Respective Lewis Acid–Base Adduct **1-CHO**

Solution structure analysis of **1-THF** dissolved in THF-*d*_8_ was carried out by using diffusion-ordered spectroscopy (DOSY). The respective 2D DOSY NMR spectrum is shown in Figure S1, and the normalized log *D*_*x*_ value of −9.2145 g mol^–1^ indicated the presence of a trinuclear aggregate [ZnMg_2_(μ_3_-OH)(O_2_CPh)_5_(THF)_3_] (**1′-THF**). The trinuclear structure of **1′-THF** was assumed to be an asymmetrical hydroxy-bridged ZnMg_2_ carboxylate cluster. Within the trinuclear ZnMg_2_(μ_3_-OH) unit, both Mg atoms and one Mg/Zn pair could have been connected by two μ-1,2-carboxylate bridges, and the second Mg/Zn pair could have been connected by just one μ-1,2-carboxylate bridge. Thus, the cluster is supposed to contain the 4-coordinated Zn atom and two nonequivalent 6-coordinated Mg centers, with their octahedral coordination completed by the solvating THF molecules. Remarkably, upon crystallization from THF, the trinuclear clusters **1′-THF** self-associate to the hexanuclear aggregate **1-THF**. It crystallizes as a heteronuclear Zn^II^_2_/Mg^II^_4_ cluster in the *P*2_1_/*c* (No. 14) space group with four uncoordinated THF molecules in the crystal lattice. The molecular structure of **1-THF** is shown in Figure S6, and the principal geometric parameters are listed in Table S1. The structure of **1-THF** features a dimeric arrangement that can be envisaged as an associate of two unsymmetrical heterometallic [ZnMg_2_(μ_3_-OH)(O_2_CPh)_5_] clusters. The trinuclear [ZnMg_2_(μ_3_-OH)] motifs are seamed by the centrosymmetrically related 6-fold Mg atoms, which are interconnected by five benzoate ligands of three different carboxylate binding modes: μ_3_-η^2^:η^1^, syn,syn-μ^2^-η^1^:η^1^, and syn,anti-μ^2^-η^1^:η^1^. The terminal Mg atoms possess an octahedral geometry, with the fifth and sixth coordination sites occupied by THF molecules. Remarkably, while Zn_4_(μ_4_-O)(O_2_CR)_6_]-type clusters can easily form extended coordination environments with THF and H_2_O molecules without breaking of the Zn–O_carboxylate_ bonds,^18d^ the Zn atoms in **1-THF** retain a distorted tetrahedral geometry despite the presence of free THF molecules in the crystal lattice. This observation indicates significant dichotomy in the coordination abilities of the Zn and Mg centers in the title heterometallic system.

The heteronuclear cluster **1-THF** comprises a unique zinc–magnesium carboxylate scaffold with incorporated bridged hydroxides and labile THF molecules coordinated to each terminal Mg atom. So, we were curious about its catalytic activity in the CHO/CO_2_ copolymerization. Remarkably, dissolution and recrystallization of **1-THF** in CHO at ambient temperature resulted in the isolation of a novel Lewis acid–base adduct between the hexanuclear zinc–magnesium cluster and CHO, [Zn_2_Mg_4_(μ_3_-OH)_2_(O_2_CPh)_10_(CHO)_4_] (**1-CHO**; for a control experiment at elevated temperature vide infra). The identity of **1-CHO** was confirmed by NMR, IR, and X-ray diffraction analysis (Figures S2–S8). The bulk purity in each case was confirmed by powder X-ray diffraction (PXRD; Figures S11–S13) and elemental analysis. The **1-CHO** adduct is isostructural with the THF analogue, and a comparison of the principal geometric parameters for **1-CHO** and **1-THF** revealed a very minor difference between them (Tables S2–S3 and Figure S9). For example, the only terminal 6-fold Mg atoms coordinate CHO molecules, and the observed average Mg–O_CHO_ (2.134 Å) and Mg–O_THF_ (2.139 Å) distances remains almost identical. Moreover, although **1-CHO** crystallizes with two CHO molecules in the crystal lattice, these CHO molecules do not coordinate to the 4-coordinated Zn centers. Interestingly, the average distances for Zn–Mg (3.433 Å) and Mg–Mg (3.425 Å) in **1-THF** (Figure S10) are within the range proposed as optimal for dinuclear ROCOP catalysis.^4^ Although a few structures of metal epoxide complexes have been reported,^11^ to the best of our knowledge, the **1-CHO** adduct is the only structurally characterized example of a heteronuclear cluster containing metal–epoxide binding.

In the next step, we tested its activity in CHO/CO_2_ copolymerization reactions at 80 °C and 1 bar of CO_2_ pressure. Notably, while some zinc and magnesium complexes bearing M–OH species are prone to react with CO_2_,^12^**1-THF** was found to be inert toward CO_2_ despite the presence of a [ZnMg_2_(μ_3_-OH)] unit. In turn, the studied system exhibited a relatively moderate catalytic activity [turnover frequency (TOF) = 30 h^–1^], affording the alternating poly(cyclohexene carbonate). The observed catalytic activity is comparable to that of a dinuclear Mg catalyst reported by Ding et al. (TOF = 43 h^–1^)^10b^ or a dinuclear zinc–magnesium catalyst (TOF = 34 h^–1^) developed by Williams et al.^8a^ and higher than that of a novel bimetallic zinc acetate catalyst (TOF = 5–6 h^–1^).^6d^ The resulting polymer featured number-average molecular weight values of 4000–5000 g mol^–1^ and showed relatively wide dispersity distributions (Table S5). This might be due to the formation of both short as well as some long chains at different metal centers or due to any postreactions as described elsewhere.^20^ Remarkably, polymerization takes place without detectable ether linkages, indicating excellent CO_2_ uptake (97%), and the reaction was highly selective toward polycarbonates, with only a trace amount of cyclic carbonate byproduct detected in the NMR spectrum. In addition, to better understand the catalyst and substrate interaction, we also studied the polymerization kinetics, applying different catalyst concentrations at 80 °C and 1 bar of CO_2_ pressure (Table S6). ^1^H NMR spectroscopy was used to monitor the reactions and obtain the initial rate coefficient, *k*_obs_ (Figures 1 and S15–S18). For different catalyst loadings, the linear plots of ln([CHO]_0_/[CHO]_t_) versus varying time illustrated a first-order dependence on the CHO concentration (Figure 1). The catalyst order of 1.07 was deduced from the slope of the ln(*k*_obs_) versus ln [cat] plot (Figure S19). Furthermore, the almost identical conversions under varied CO_2_ pressures indicated a zero-order dependence on the CO_2_ concentration (Figure S20).

**Figure 1:**
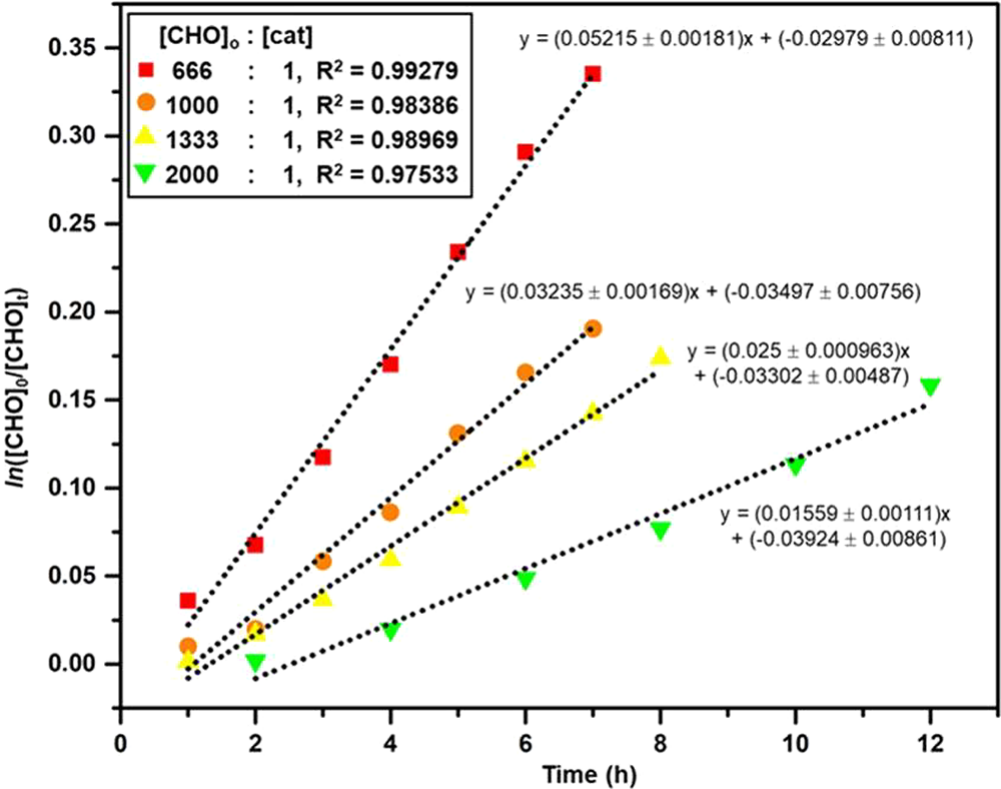
Plots to determine the initial rates depending on [**1-THF**], using ^1^H NMR analysis of aliquots taken at defined intervals. The dotted lines indicate linear fits, which are represented by their corresponding line equations. All experiments were conducted at 80 °C and 1 bar of CO_2_ with 8 M CHO in diethyl carbonate (Table S6). The data points indicate the average values for three consecutive experiments in each case.

On the basis of the acquired structural characteristics, kinetic studies, and the observed dichotomy in the coordination abilities of Zn and Mg centers, we draw a plausible mechanism of the copolymerization reaction, as shown in Figure 2. It is reasonable to assume that the trinuclear zinc–magnesium hydroxide carboxylate acts as the initiator. The epoxide activation occurs at the more Lewis acidic 4-fold Mg center, whereas the Zn ion plays a supporting role in the catalytic cycle. At ambient temperature, the putative CHO adduct **I** is relatively stable; however, higher temperature introduces a significant flexibility to the core structure and promotes the nucleophilic attack of the carboxylate on the coordinated CHO (transient state **II**) to give the zinc–magnesium carboxylate–alkoxide trinuclear catalytic system **III**. This event is considered to be a rate-determining step of the copolymerization reaction. The resulting primary insertion product enters the catalytic cycle with an intermediate carbonate species **IV**. The propagation cycle continues with CO_2_ insertion into the Mg–alkoxide bond and nucleophilic attack of the carbonate species on the coordinated CHO molecule, and the polymerization cycle can continue to harness the alternating poly(cyclohexene carbonate). The putative catalytically active heterotrinuclear Zn^II^/Mg^II^_2_ aggregate incorporates two Mg centers with a possibility to generate one or two growing chains; however, the number of polymer chains calculated per Mg atom was found to be ca. 1 (Table S5; for simplicity, only one growing chain is shown in Figure 2).

**Figure 2:**
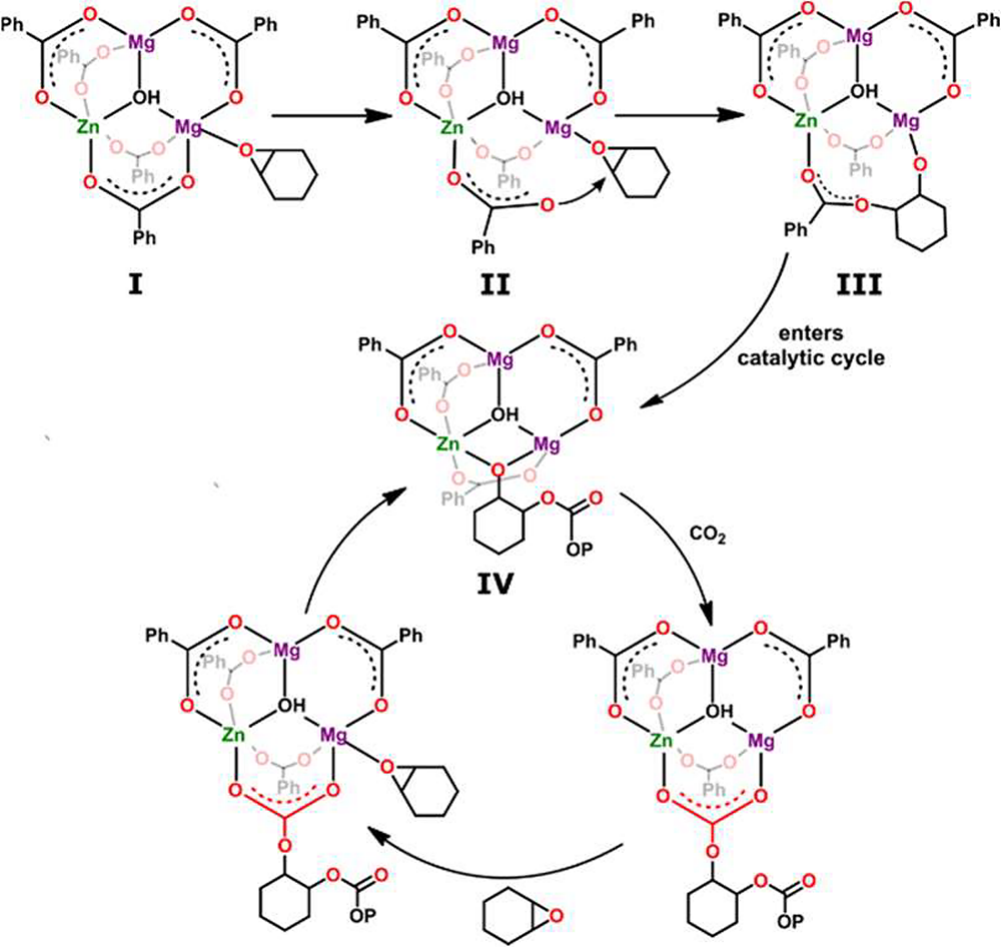
Proposed catalytic cycle for the ROCOP.

To properly compare and understand the significance of a heterodinuclear synergic relationship, several synthetic strategies to place different metal centers in the same chemical environment using symmetrical macrocyclic ligands have been developed over the past few years. We demonstrated the efficient synthesis of novel multinuclear zinc–magnesium heterometallic frameworks just supported by a simple carboxylate ligand and exploited their different Lewis acidities in the copolymerization of CO_2_ and CHO. Remarkably, the studies led to the isolation and structural characterization of the relatively stable Lewis acid–base adduct of CHO with the hexanuclear zinc–magnesium hydroxide benzoate cluster, **1-CHO**, which represents the first well-defined epoxide adduct with a heterometallic complex. The crystal structure analysis of **1-CHO** revealed that CHO molecules only interact with the higher coordinated Mg centers, while the 4-coordinated Zn centers remain intact, which nicely demonstrates the dichotomy in the coordination abilities of the Zn and Mg centers in heterometallic carboxylate clusters. Overall, our results open up new avenues of research relevant to the polymerization and copolymerization reactions initiated by heterometallic systems, and they can aid in the engineering of novel homogeneous and heterogeneous catalytic systems.

## Supporting Information Available

General considerations and synthetic procedures, characterization of compounds including typical figures, NMR and FTIR spectra, PXRD pattern, and X-ray crystal structure data, and catalysis and kinetic data for ROCOP (PDF)

## References

[ref1] aChilders M. I.; Longo J. M.; Van Zee N. J.; LaPointe A. M.; Coates G. W. Stereoselective Epoxide Polymerization and Co-polymerization. Chem. Rev. 2014, 114, 8129–8152. 10.1021/cr400725x. 25007101

[ref2] aCoates G. W.; Moore D. R. Discrete Metal-Based Catalysts for the Co-polymerization of CO2 and Epoxides: Discovery, Reactivity, Optimization, and Mechanism. Angew. Chemie Int. Ed. 2004, 43, 6618–6639. 10.1002/anie.200460442. 15558659

[ref3] Inoue S.; Koinuma H.; Tsuruta T. Co-polymerization of Carbon Dioxide and Epoxide. J. Polym. Sci. Part B Polym. Lett. 1969, 7, 287–292. 10.1002/pol.1969.110070408.

[ref4] aLu X.-B.; Darensbourg D. J. Cobalt Catalysts for the Coupling of CO_2_ and Epoxides to Provide Polycarbonates and Cyclic Carbonates. Chem. Soc. Rev. 2012, 41, 1462–1484. 10.1039/C1CS15142H. 21858339

[ref5] aNozaki K.; Nakano K.; Hiyama T. Optically Active Polycarbonates: Asymmetric Alternating Copolymerization of Cyclohexene Oxide and Carbon Dioxide. J. Am. Chem. Soc. 1999, 121, 11008–11009. 10.1021/ja992433b.

[ref6] aCheng M.; Lobkovsky E. B.; Coates G. W. Catalytic Reactions Involving C1 Feedstocks: New High-Activity Zn(II)-Based Catalysts for the Alternating Co-polymerization of Carbon Dioxide and Epoxides. J. Am. Chem. Soc. 1998, 120, 11018–11019. 10.1021/ja982601k.

[ref7] aNagae H.; Aoki R.; Akutagawa S.; Kleemann J.; Tagawa R.; Schindler T.; Choi G.; Spaniol T. P.; Tsurugi H.; Okuda J.; Mashima K. Lanthanide Complexes Supported by a Trizinc Crown Ether as Catalysts for Alternating Copolymerization of Epoxide and CO_2_: Telomerization Controlled by Carboxylate Anions. Angew. Chem., Int. Ed. 2018, 57, 2492–2496. 10.1002/anie.201709218. 29292563

[ref8] aGarden J. A.; Saini P. K.; Williams C. K. Greater than the Sum of Its Parts: A Heterodinuclear Polymerization Catalyst. J. Am. Chem. Soc. 2015, 137, 15078–15081. 10.1021/jacs.5b09913. 26618526

[ref9] Deacy A. C.; Kilpatrick A. F. R.; Regoutz A.; Williams C. K. Understanding Metal Synergy in Heterodinuclear Catalysts for the Co-polymerization of CO_2_ and Epoxides. Nat. Chem. 2020, 12, 372–380. 10.1038/s41557-020-0450-3. 32221501

[ref10] aMoore D. R.; Cheng M.; Lobkovsky E. B.; Coates G. W. Mechanism of the Alternating Co-polymerization of Epoxides and CO_2_ Using β -Diiminate Zinc Catalysts: Evidence for a Bimetallic Epoxide Enchainment. J. Am. Chem. Soc. 2003, 125, 11911–11924. 10.1021/ja030085e. 14505413

[ref11] aDarensbourg D. J.; Holtcamp M. W.; Khandelwal B.; Klausmeyer K. K.; Reibenspies J. H. Syntheses and Structures of Epoxide Adducts of Soluble Cadmium(II) Carboxylates. Models for the Initiation Process in Epoxide/CO_2_ Coupling Reactions. J. Am. Chem. Soc. 1995, 117, 538–539. 10.1021/ja00106a065.

[ref12] aAppel A. M.; Bercaw J. E.; Bocarsly A. B.; Dobbek H.; DuBois D. L.; Dupuis M.; Ferry J. G.; Fujita E.; Hille R.; Kenis P. J. A.; Kerfeld C. A.; Morris R. H.; Peden C. H. F.; Portis A. R.; Ragsdale S. W.; Rauchfuss T. B.; Reek J. N. H.; Seefeldt L. C.; Thauer R. K.; Waldrop G. L. Frontiers, Opportunities, and Challenges in Biochemical and Chemical Catalysis of CO_2_ Fixation. Chem. Rev. 2013, 113, 6621–6658. 10.1021/cr300463y. 23767781 PMC3895110

[ref13] Mbabazi R.; Wendt O. F.; Allan Nyanzi S.; Naziriwo B.; Tebandeke E. Advances in Carbon Dioxide and Propylene Oxide Co-polymerization to Form Poly(Propylene Carbonate) over Heterogeneous Catalysts. Results Chem. 2022, 4, 100542 10.1016/j.rechem.2022.100542.

[ref14] Ree M.; Bae J. Y.; Jung J. H.; Shin T. J. A New Co-polymerization Process Leading to Poly(Propylene Carbonate) with a Highly Enhanced Yield from Carbon Dioxide and Propylene Oxide. J. Polym. Sci. Part A Polym. Chem. 1999, 37, 1863–1876. 10.1002/(SICI)1099-0518(19990615)37:12<1863::AID-POLA16>3.0.CO;2-K.

[ref15] aQin Y.; Wang X. Carbon dioxide-based copolymers: Environmental benefits of PPC, an industrially viable catalyst. Biotechnol. J. 2010, 5, 1164–1180. 10.1002/biot.201000134. 21058318

[ref16] aMeng Y. Z.; Du L. C.; Tiong S. C.; Zhu Q.; Hay A. S. Effects of the structure and morphology of zinc glutarate on the fixation of carbon dioxide into polymer. J. Polym. Sci. A Polym. Chem. 2002, 40, 3579–3591. 10.1002/pola.10452.

[ref17] aChisholm M. H.; Navarro-Llobet D.; Zhou Z. Poly(Propylene Carbonate). 1. More about Poly(Propylene Carbonate) Formed from the Co-polymerization of Propylene Oxide and Carbon Dioxide Employing a Zinc Glutarate Catalyst. Macromolecules 2002, 35, 6494–6504. 10.1021/ma020348+.

[ref18] aLewiński J.; Zachara J.; Horeglad P.; Glinka D.; Lipkowski J.; Justyniak I. Structural Evidence of the Epoxide Oxygen Propensity to Double Coordination. Inorg. Chem. 2001, 40, 6086–6087. 10.1021/ic015572e. 11703104

[ref19] aLewiński J.; Bury W.; Dutkiewicz M.; Maurin M.; Justyniak I.; Lipkowski J. Alkylzinc Carboxylates as Efficient Precursors for Zinc Oxocarboxylates and Sulfidocarboxylates. Angew. Chem. Inter. Ed. 2008, 47, 573 10.1002/anie.200703125. 18034439

[ref20] Liu B.; Chen L.; Zhang M.; Yu A. Degradation and Stabilization of Poly(Propylene Carbonate). Macromol. Rapid Commun. 2002, 23, 881 10.1002/1521-3927(20021001)23:15<881::AID-MARC881>3.0.CO;2-C.

