# Supplementary material for: Multinuclear Zinc–Magnesium Hydroxide Carboxylates: A Predesigned Model System for Copolymerization of CO2 with Epoxides
Source: Inorg Chem. 2023 Sep 15;62(40):16274–9. doi: 10.1021/acs.inorgchem.3c02177 (PMC10565889; doi:10.1021/acs.inorgchem.3c02177)
Supplement: Supplementary file 1 — ic3c02177_si_001.pdf [file ic3c02177_si_001.pdf]

Supporting Information

Multinuclear Zinc-Magnesium Hydroxide Carboxylates: A Predesigned Model System for Co-polymerization of CO_2 with Epoxides

Vijay Gupta[a], Iwona Justyniak[b], Elżbieta Chwojnowska[b], Vadim Szejko[a], and Janusz Lewiński[a,b]*

[a]*Faculty of Chemistry, Warsaw University of Technology, Noakowskiego 3, 00-664 Warsaw, Poland;*
[b]*Institute of Physical Chemistry, Polish Academy of Sciences, Kasprzaka 44/52, 01-224 Warsaw, Poland.*

*Author for correspondence:
Prof. Janusz Lewiński, E-mail: janusz.lewinski@pw.edu.pl

- **General considerations and Synthetic procedures**
- **Diffusivity measurement**
- **^{1}H NMR studies**
- **FTIR studies**
- **X-ray Diffraction Studies**
- **Kinetics study for ROCOP**

General Considerations. Unless otherwise stated, all manipulations involving air and moisture-sensitive organometallic compounds were conducted under a dry, oxygen-free argon atmosphere either using standard Schlenk techniques or in a glovebox (MBraun UniLab Plus; < 0.1 ppm O_2, < 0.1 ppm H_2O). All glassware was stored in a 150°C oven overnight before use. All reagents were purchased from commercial vendors: benzoic acid (ABCR), diethyl zinc (ABCR), n-butyl magnesium (1M solution in heptane) (sigma) and used as received. Solvents were purified by passage through activated aluminium oxide (MBraun SPS) and stored over 3Å molecular sieves. The deuterated solvents were dried over Na/K, distilled under an argon atmosphere before use, and stored over molecular sieves. Cyclohexene oxide (CHO) was obtained from ABCR and prior to its use it was distilled from CaH_2, thoroughly degassed and stored under argon. Research grade carbon dioxide was used for copolymerization reactions.

Synthetic Procedures

Initial reaction to obtain heteronuclear Zn/Mg oxide/hydroxide carboxylates. Et_2Zn (2.0 M in hexane, 1 mL, 2 mmol) and di-n-butylmagnesium (1.0 M in heptane, 2 mL, 2 mmol) was added dropwise to a solution of benzoic acid (0.73 g, 6 mmol) in THF (30 mL) at –78 °C. The mixture was warmed to room temperature, and stirred for 8 h. Afterwards, to the vigorously stirred reaction degassed water (18 µL, 1 mmol) was added and the reaction mixture was stirred for another 24 h. A mixture of the product was isolated as colourless crystals after filtration and crystallization in THF-hexane at 0-5 °C. However, it was not possible to separate the different compounds; but we were fortunate enough to get the crystal structure of two different compounds from the batch of crystals.

General Procedure for the synthesis of 1-THF. Et_2Zn (2.0 M in hexane, 1 mL, 2 mmol) and di-n-butylmagnesium (1.0 M in heptane, 4 mL, 4 mmol) was added dropwise to a solution of benzoic acid (1.23 g, 10 mmol) in THF (30 mL) at –78 °C. The mixture was warmed to room temperature, and stirred for 8 h. Afterwards, to the vigorously stirred reaction degassed water (36 µL, 2 mmol) was added and the reaction mixture was stirred for another 24 h. The product was isolated as colourless crystals after filtration and crystallization in THF-hexane at 0-5 °C (yield = 65%, 1.352 g). Elemental analysis: calcd (%) for **1-THF·4 THF** ($C_{102}H_{116}O_{30}Mg_4Zn_2$) (2056.02): C, 59.76; H, 5.70. Found (%): C, 58.92; H, 6.02.

General procedure for the synthesis of 2. Di-n-butylmagnesium (1.0 M in heptane, 3 mL, 3 mmol) was added dropwise to a solution of benzoic acid (740 mg, 6 mmol) in THF (20 mL) at –78 °C. The mixture was warmed to room temperature and allowed to stir for overnight. The product was isolated as colourless crystals after filtration and crystallization in THF-hexane at 0-5 °C (yield = 70%, 815 mg). Elemental analysis: calcd (%) for **2·0.5 THF** ($C_{62}H_{70}O_{17}Mg_3$) (1160.12): C, 64.19; H, 6.08. Found (%): C, 64.52; H, 6.74.

General procedure for the ROCOP. Cyclohexene oxide (2 mL, 20 mmol) and the catalyst (0.1 mol%) were added to a Schlenk tube in the glovebox. This Schlenk tube was then subjected to five rapid vacuum/CO_2 (pressure regulated to 1 bar) cycles, before it was left stirring under 1 atm CO_2, at 80 °C in an oil bath. Aliquots were taken under a positive pressure of CO_2. Reaction was quenched by cooling the sample and exposing it to air. ^{1}H NMR spectra were taken, in air and in $CDCl_3$, before the crude product was obtained through removal of volatile CHO under vacuum. GPC analysis was carried out on the crude sample which was re-dissolved in dichloromethane for analysis.

Diffusivity measurement

The DOSY spectra were acquired on Bruker AVANCE II 300 MHz spectrometer at 298 K. Pulsed field gradient double stimulated echo convection-compensated (PFGSTE) sequence with total of 16 diffusion encoding bipolar gradients (ranging from 3 to 48 G/cm, smoothed-square shaped, equal steps in gradient squared) was used and the total width of the gradient pulse was optimized to achieve attenuation of about 90% of the initial intensity of the signals. Overall, the key acquisition parameters were as follows: total length of gradient encoding pulses gradient - 2ms, diffusion delay - 150ms, gradient recovery delay - 0.1ms, relaxation delay - 2.8s. Steady-state scans in number of 4 were performed prior to acquisition of the data. Raw data was processed with powerful DOSY Toolbox which is extensively described in its author's paper.[S1] Samples were dissolved in dry and degassed THF-d8 at concentration ca. 15mM. The molecular masses of analyzed compounds were estimated utilizing an external calibration curve (ECC) approach with normalized diffusion coefficients, with 9-methylanthracene (9-MeA; MW = 192) as an internal reference.[S2, S3] Moreover, we have applied the van-der-Waals radii-based correction in order to account for the underestimation of MW due to the presence of heavy atoms, according to the literature methods.[S4]

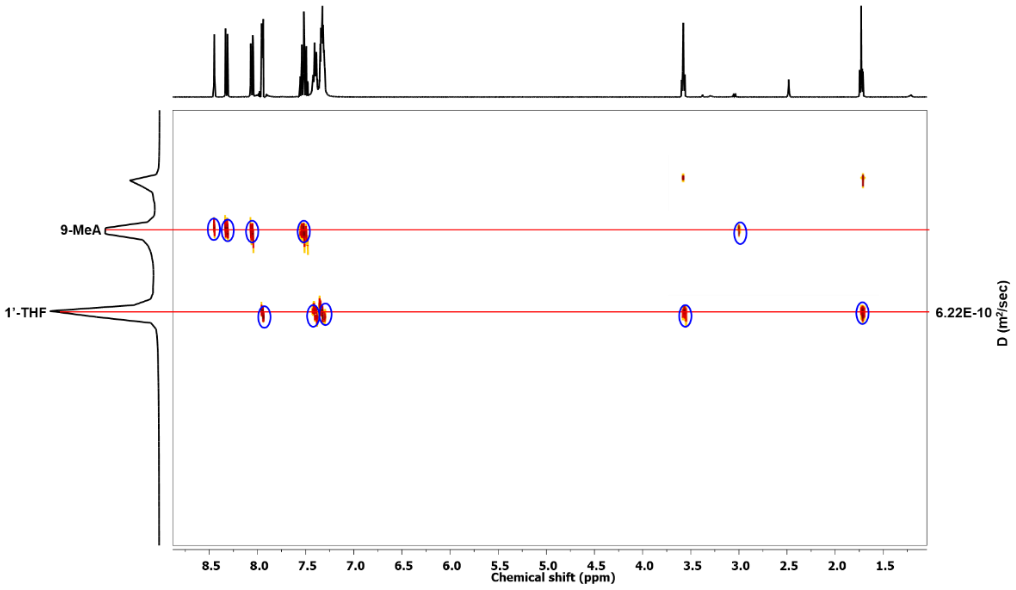

Solvent = THF				
Reference = 9-Methylanthracene				
log$D_{x,norm}$ = -9.2145 g/mol				
MW_{calc} = 955 g/mol				
			**Within expected error interval**	
**ECC**	$\boldsymbol{MW_{det}}$	$\boldsymbol{MW_{dif}}$	**Empirical**	**Theoretical**
**CS**	965 g/mol	-1%	✓	✓
**Merge**	831 g/mol	15%	✓	
**DSE**	753 g/mol	27%		
**ED**	610 g/mol	57%		
$MD_w = 5.15\cdot10^{29}$ g/(mol·m³) okay				

where, MW_{cal} and MW_{det} represents the molecular weight calculated and molecular weight determined using the external calibration curve (ECC) method from the DOSY NMR analysis considering the molecular models as compact spheres (CS), dissipated spheres and ellipsoids (DSE), expanded discs (ED), or merged model, respectively. MW_{dif} represents the difference between MW_{cal} and MW_{det}.

Figure S1. 2D DOSY-NMR spectrum of **1-THF** in d_8-THF and data for molecular weight estimation.

^{1}H NMR studies

NMR spectra were acquired on a Bruker 300 MHz spectrometer at 298 K.

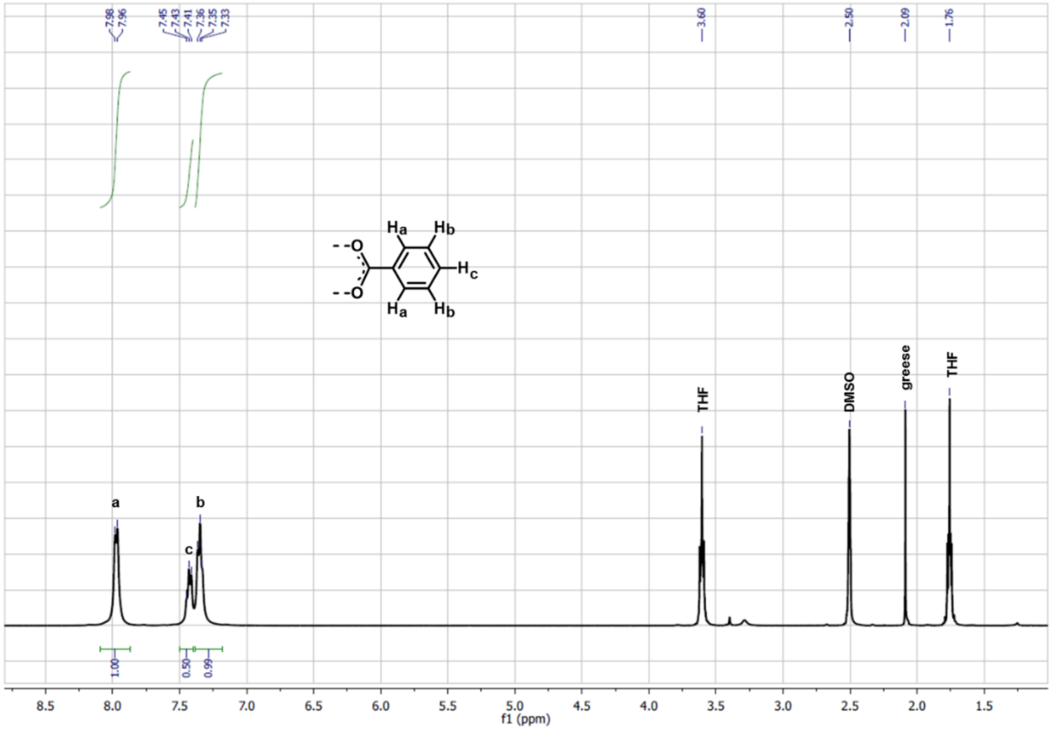

Figure S2. ^{1}H NMR spectrum for **1-THF** in DMSO-d_6.

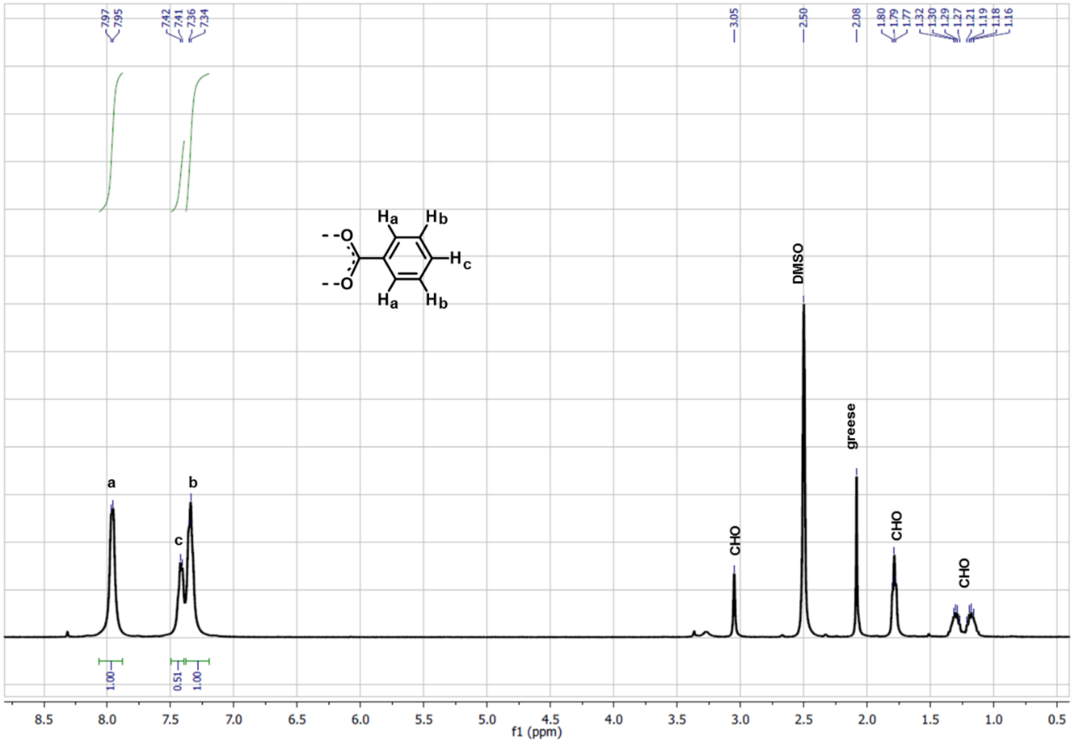

Figure S3. ^{1}H NMR spectrum for **1-CHO** in DMSO-d_6.

FTIR studies

FTIR spectra were measured with a Bruker Tensor II spectrometer using the ATR technique.

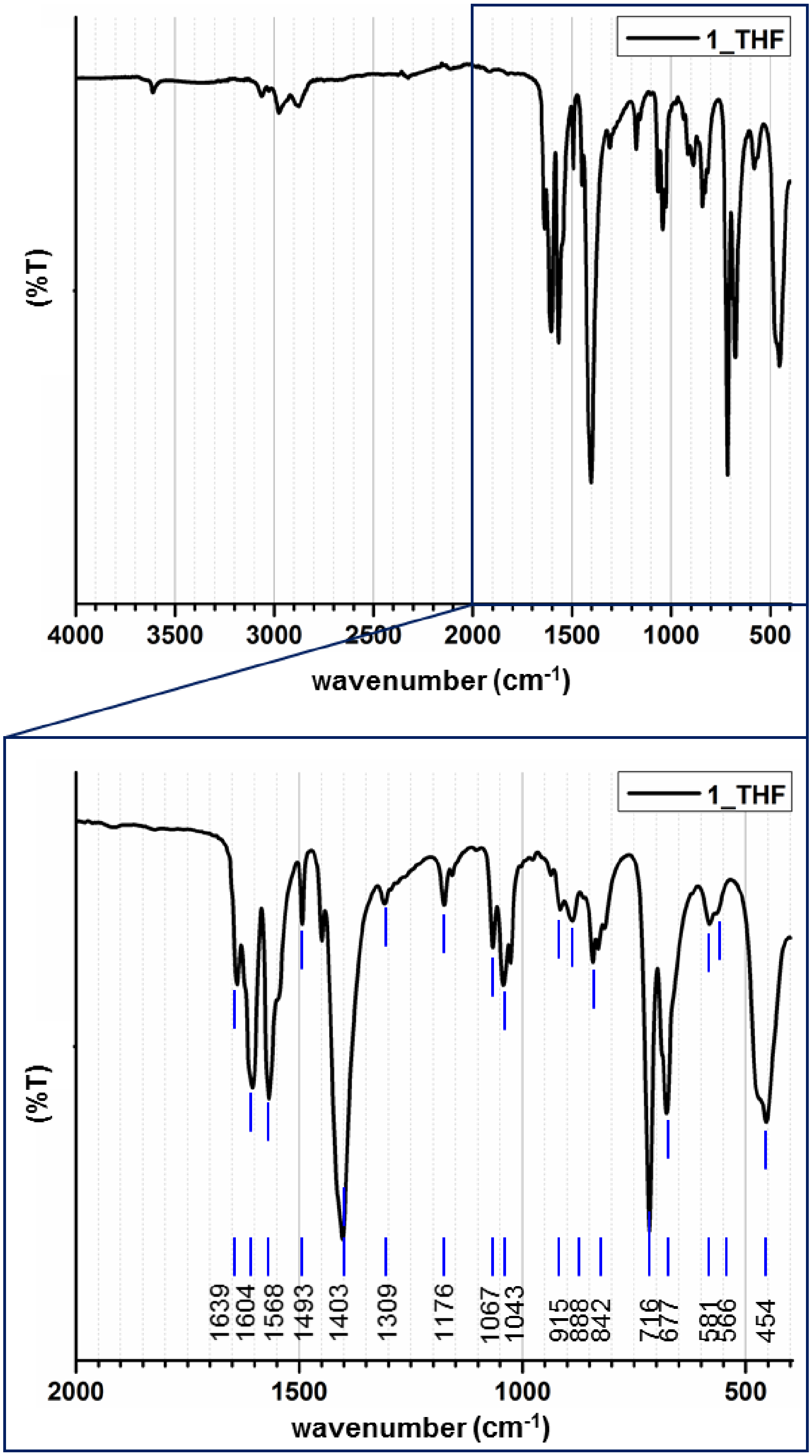

Figure S4. FTIR spectrum for **1-THF**.

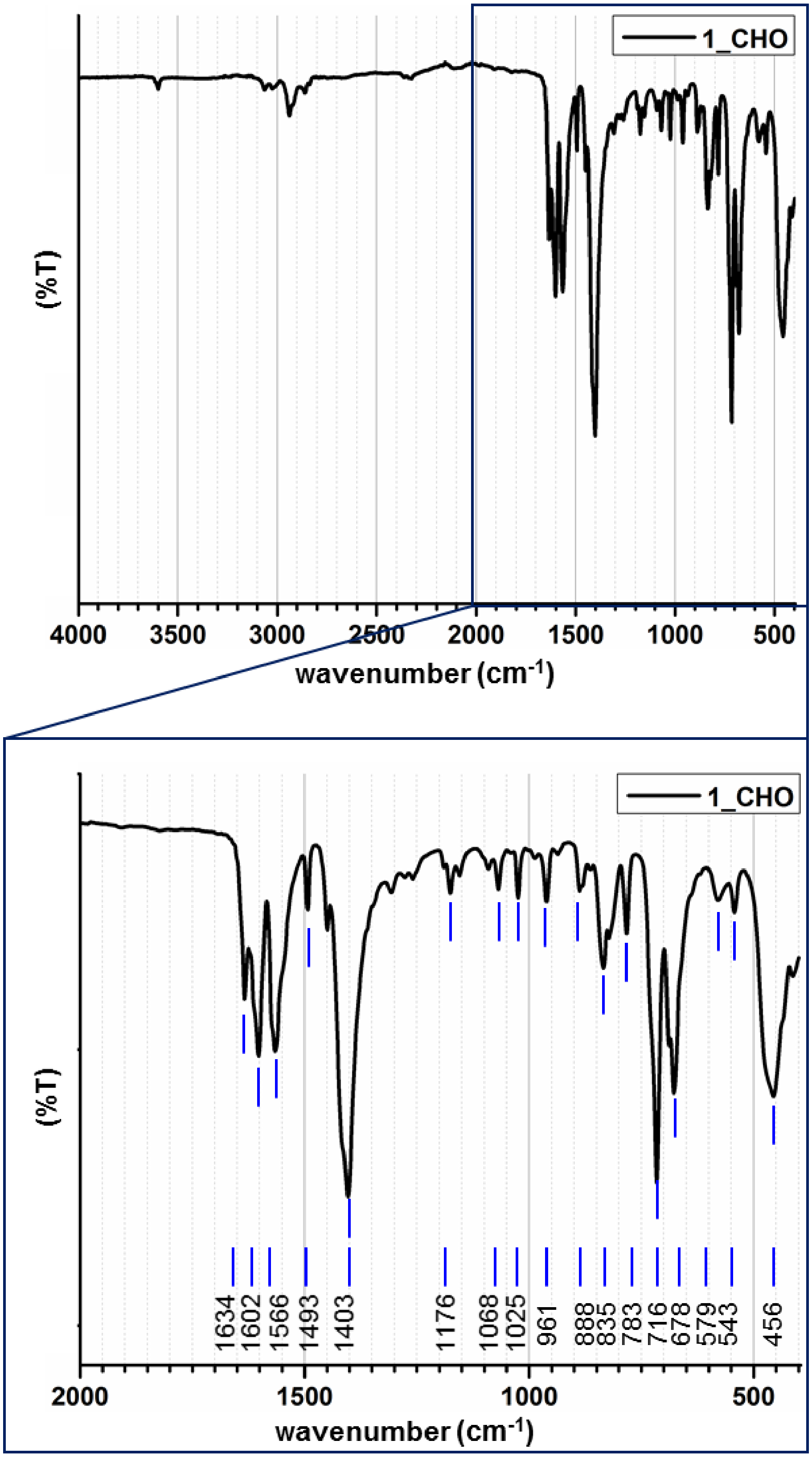

Figure S5. FTIR spectrum for **1-CHO**.

X-ray Diffraction Studies

The single crystal X-ray data for complexes **1-THF**, **1-CHO** and **2** were collected at 100(2)K on a SuperNova Agilent diffractometer using graphite monochromated MoKα radiation (λ = 0.71073 Å) for **1-THF**, **2** and CuKα radiation (λ = 1.54184 Å) for **1-CHO**. The crystals of all complexes were selected under Paratone-N oil, mounted on the nylon loops, and positioned in the cold stream on the diffractometer. The data were processed with CrysAlisPro.[S5] The structures **1-THF**, **1-CHO** and **2** were solved by direct methods using the SHELXT program and were refined by full matrix least–squares on F^2 using the program SHELXL.[S6] All non-hydrogen atoms were refined with anisotropic displacement parameters. Hydrogen atoms were added to the structure model at geometrically idealized coordinates and refined as riding atoms.

Crystallographic data (excluding structure factors) for the structure reported in this paper have been deposited with the Cambridge Crystallographic Data Centre as a supplementary publication. Copies of the data can be obtained free of charge on application to CCDC, 12 Union Road, Cambridge CB21EZ, UK (fax: (+44)1223-336-033; e-mail: deposit@ccdc.cam.ac.uk). CCDC: 2239055 (**1-THF**), 2239056 (**1-CHO**), 2240656 (**2**).

Powder XRD data were collected on a PANalytical Empyrean diffractometer. Measurements employed Ni-filtered Cu Ka radiation of a copper sealed tube charged with 40 kV voltage and 40 mA current in a Bragg–Brentano geometry with a beam divergence of 1 deg. in the scattering plane. The sample was spread over the surface of a porous glass plate fixed to the sample holder. Diffraction patterns were measured in the scattering angle range of 3–50 degrees by step scanning in steps of 0.02 degree.

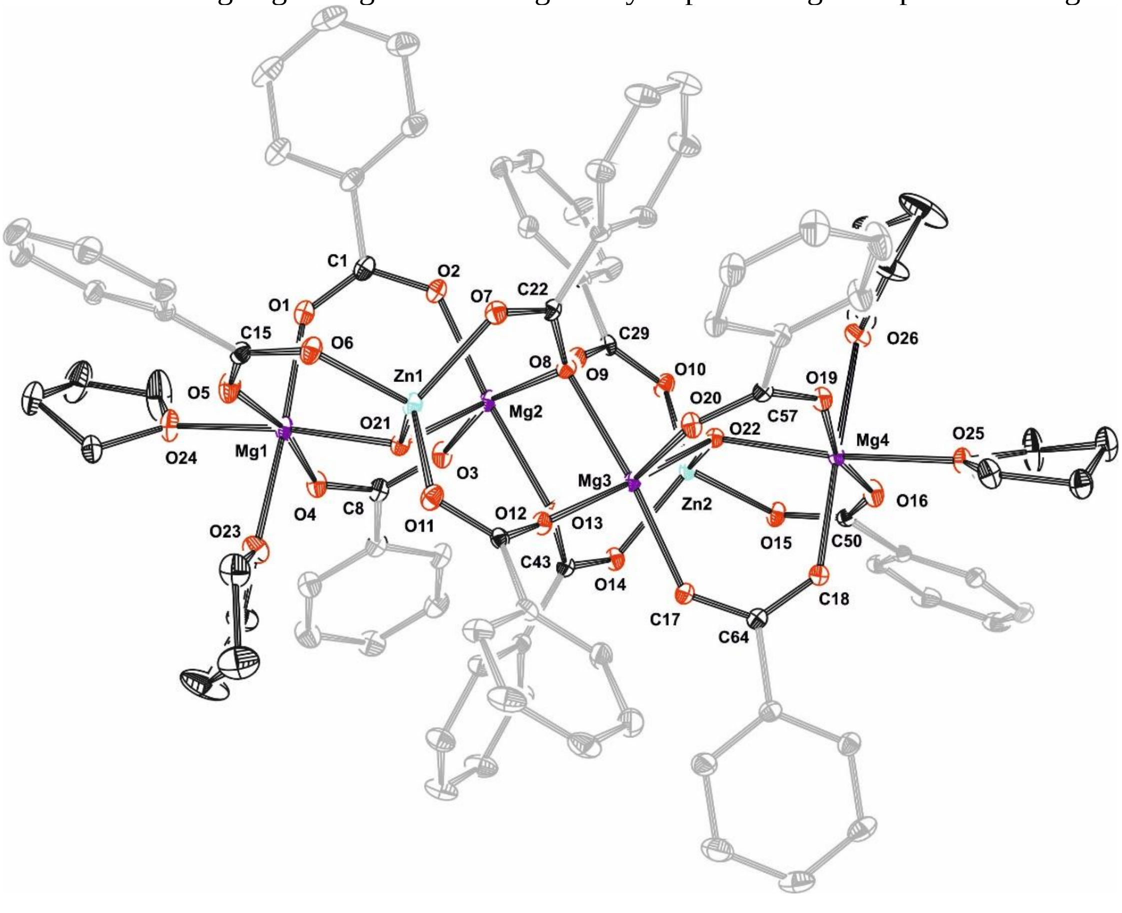

Figure S6. The molecular structure of **1-THF** with thermal ellipsoids is set at 30% probability. Hydrogen atoms and lattice THF molecules have been omitted for clarity.

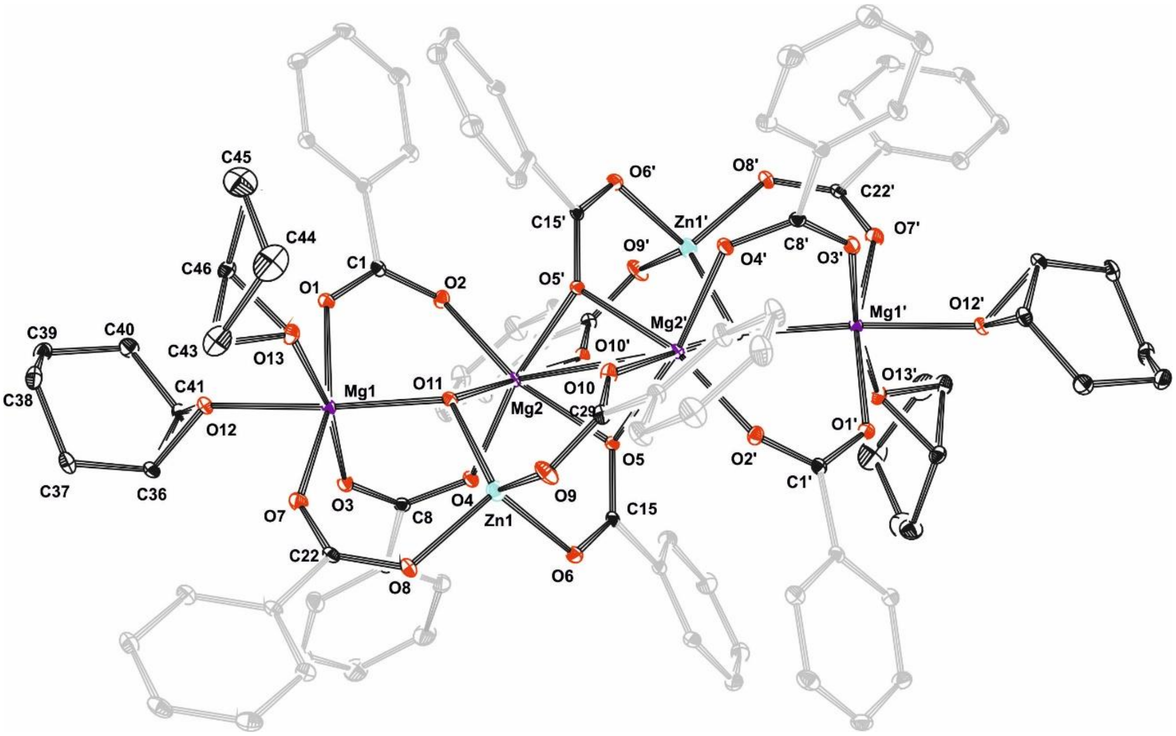

Figure S7. The molecular structure of **1-CHO** with thermal ellipsoids is set at 30% probability. Hydrogen atoms and lattice CHO molecules have been omitted for clarity. Symmetry transformations used to generate equivalent atoms: (*-x+1,-y+1,-z+1*).

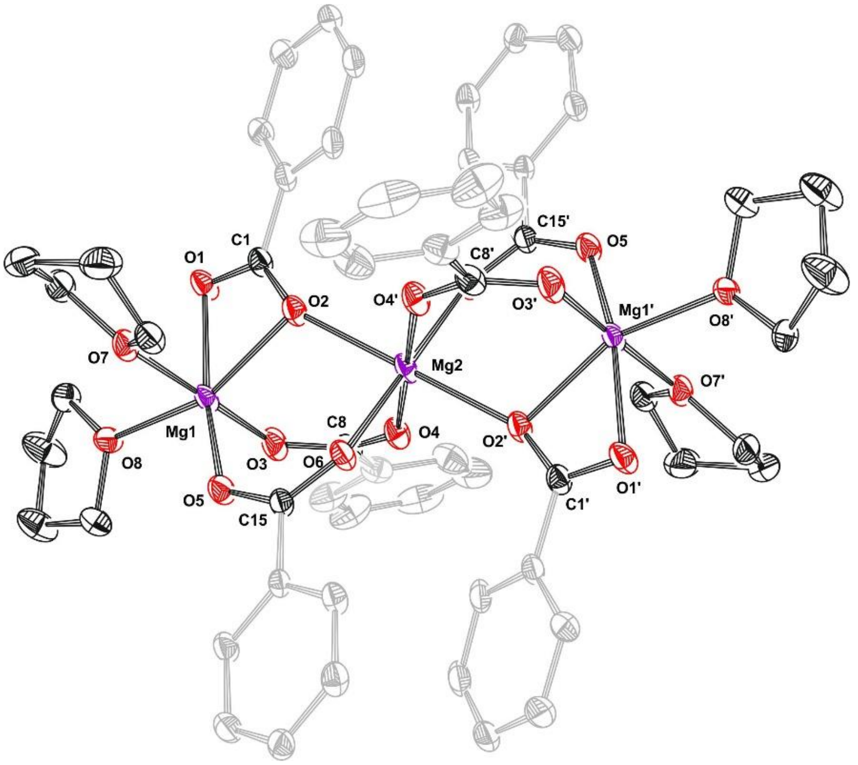

Figure S8. The molecular structure of **2** with thermal ellipsoids is set at 30% probability. Hydrogen atoms and lattice THF molecules have been omitted for clarity. Symmetry transformations used to generate equivalent atoms: *(-x+1,-y+1,-z+1), (-x,-y,-z+2).*

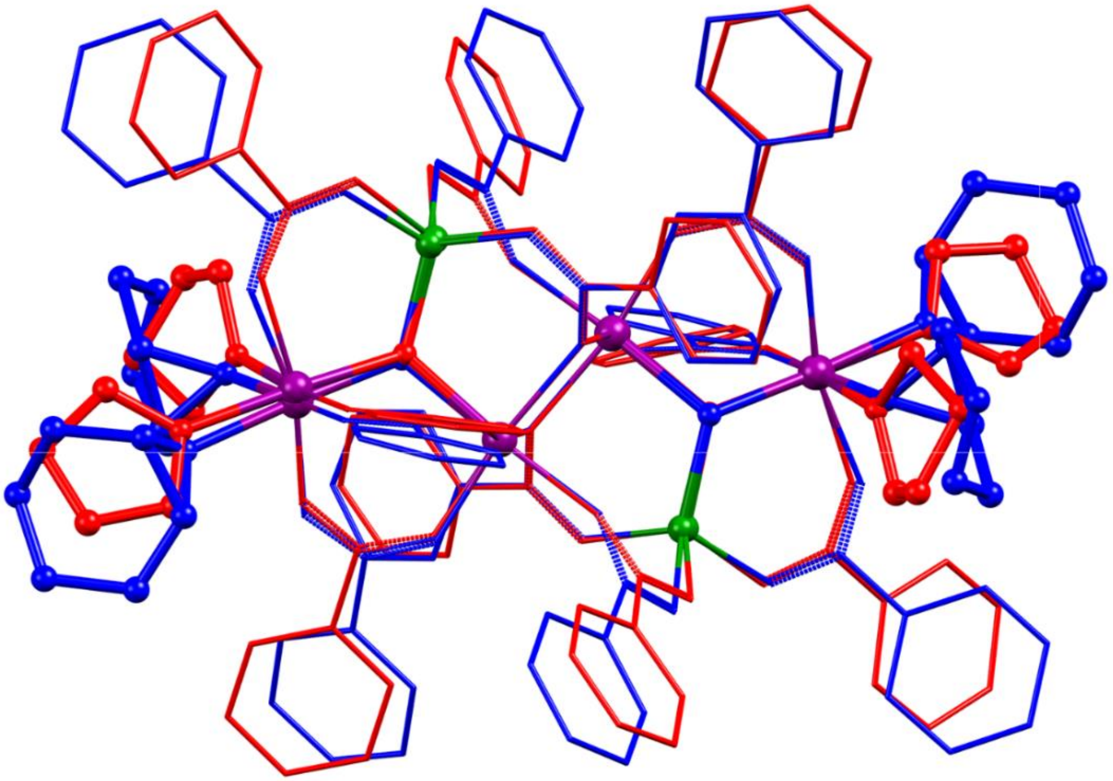

Figure S9. Structural overlay for **1-THF** (red) and **1-CHO** (blue).

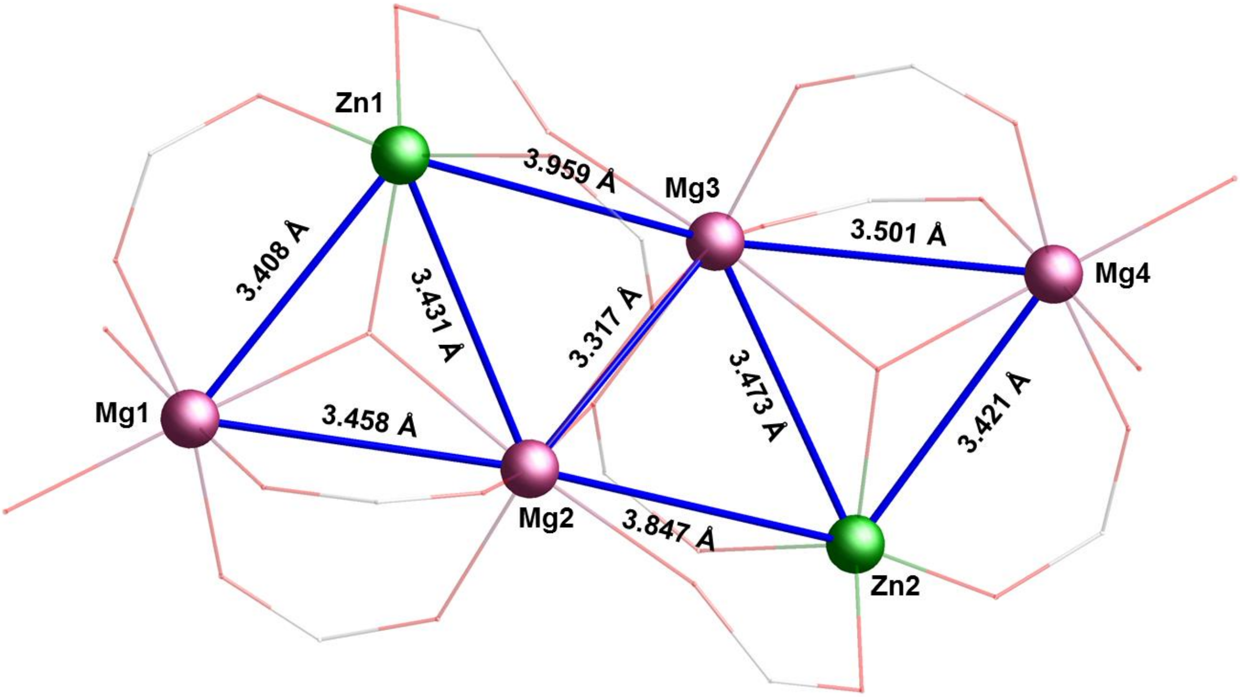

Figure S10. The distance between the metal centers in **1-THF**.

Table S1 Crystallographic data and Structure refinement parameters for 1-THF, 1-CHO and 2.

Identification code	**1-THF**	**1-CHO**	**2**
Empirical formula	$C_{102}H_{116}Mg_4O_{30}Zn_2$	$C_{106}H_{112}Mg_4O_{28}Zn_2$	$C_{62}H_{70}Mg_3O_{17}$
Formula weight	2049.92	2061.93	1160.11
Temperature/K	100(2)	100(2)	100.(2)
Crystal system	monoclinic	triclinic	Triclinic
Space group	$P2_1/c$	*P*-1	P-1
a/Å	26.3595(4)	11.8433(4)	11.4428(2)
b/Å	14.7712(2)	13.7044(5)	11.8841(2)
c/Å	26.4995(4)	17.3991(6)	21.5849(4)
α/°	90	70.030(3)	95.889(2)
β/°	102.2240(10)	74.998(3)	90.828(2)
γ/°	90	70.535(3)	97.614(2)
Volume/Å^3	10083.9(3)	2468.72(17)	2892.87(9)
Z	4	1	2
ρ_{calc}g/cm^3	1.35	1.387	1.332
μ/mm^{-1}	0.579	1.504	0.125
F(000)	4304	1080	1228
Crystal size/mm^3	0.140 x 0.110 x 0.070	0.160 × 0.110 × 0.070	0.130 × 0.092 × 0.041
Radiation	MoKα (λ = 0.71073)	CuKα (λ = 1.54184)	MoKα (λ = 0.71073)
Theta range for data collection /°	2.098 to 26.500	2.739 to 70.244	2.435 to 27.000
Index ranges	-32 ≤ h ≤ 33, -18 ≤ k ≤ 18, -33 ≤ l ≤ 32	-14 ≤ h ≤ 14, -16 ≤ k ≤ 16, -19 ≤ l ≤ 21	-14 ≤ h ≤ 14, -15 ≤ k ≤ 15, -27 ≤ l ≤ 27
Reflections collected	74089	27497	51191
Independent reflections	20681 [R(int) = 0.0319]	9313 [R_{int} = 0.0254]	12627 [R_{int} = 0.0300]
Data/restraints/parameters	20681 / 6 / 1253	9317/0/631	12627/66/780
Goodness-of-fit on F^2	1.066	1.055	1.021
Final R indexes [I>=2σ (I)]	R_1^a = 0.0454, wR_2^b = 0.1087	R_1 = 0.0481, wR_2 = 0.1440	R_1 = 0.0617, wR_2 = 0.1494
Final R indexes [all data]	R_1 = 0.0618, wR_2 = 0.1212	R_1 = 0.0495, wR_2 = 0.1450	R_1 = 0.0774, wR_2 = 0.1606
Largest diff. peak/hole / e Å^{-3}	0.614 and -0.545	0.582/-1.682	0.69/-0.45

$^a R_1 = \Sigma||F_o| - |F_c||/\Sigma|F_o|$. $^b wR_2 = [\Sigma w(F_o^2 - F_c^2)^2/\Sigma w(F_o^2)^2]^{1/2}$, where $w = 1/[\sigma^2(F_o^2) + (aP)^2 + bP]$, $P = (F_o^2 + 2F_c^2)/3$

Table S2 Selected bond lengths (Å) and angles (deg) for **1-THF**.

Bond Lengths (Å)

Zn1-O6	1.9585(17)	Zn1-O7	1.9780(17)	Zn1-O11	1.9715(17)
Zn1-O21	1.9644(16)	Zn2-O10	1.9866(17)	Zn2-O14	1.9781(17)
Zn2-O15	1.9525(16)	Zn2-O22	1.9637(15)	Mg1-O1	2.029(2)
Mg1-O4	2.0112(18)	Mg1-O5	2.0230(19)	Mg1-O21	2.0927(18)
Mg1-O23	2.170(2)	Mg1-O24	2.1022(19)	Mg2-O2	2.0210(18)
Mg2-O3	2.0172(18)	Mg2-O8	2.1130(17)	Mg2-O9	2.0438(17)
Mg2-O13	2.1723(17)	Mg2-O21	2.1104(18)	Mg3-O8	2.1477(17)
Mg3-O12	2.0466(17)	Mg3-O13	2.1100(16)	Mg3-O17	2.0146(17)
Mg3-O20	2.0119(17)	Mg3-O22	2.1415(17)	Mg4-O16	2.0429(18)
Mg4-O18	2.0274(18)	Mg4-O19	2.0257(17)	Mg4-O22	2.0921(17)
Mg4-O25	2.1236(17)	Mg4-O26	2.1617(18)		

Bond Angles (deg)

O14-Zn2-O10	115.59(7)	O15-Zn2-O10	98.15(7)	O15-Zn2-O14	100.58(7)
O15-Zn2-O22	112.54(7)	O22-Zn2-O10	117.49(7)	O22-Zn2-O14	110.47(7)
O1-Mg1-O21	94.40(8)	O1-Mg1-O23	174.68(8)	O4-Mg1-O1	99.90(8)
O4-Mg1-O5	167.89(9)	O4-Mg1-O21	91.89(7)	O4-Mg1-O23	85.29(8)
O4-Mg1-O24	84.69(8)	O5-Mg1-O1	89.39(9)	O5-Mg1-O21	95.15(7)
O5-Mg1-O23	85.31(8)	O5-Mg1-O24	87.43(8)	O21-Mg1-O23	86.51(8)
O21-Mg1-O24	174.11(9)	O24-Mg1-O23	88.44(8)	O2-Mg2-O8	89.01(7)
O2-Mg2-O9	92.09(7)	O2-Mg2-O13	165.72(7)	O2-Mg2-O21	88.31(7)
O3-Mg2-O2	107.51(8)	O3-Mg2-O8	162.57(8)	O3-Mg2-O9	88.84(7)
O3-Mg2-O13	86.21(7)	O3-Mg2-O21	95.79(7)	O8-Mg2-O13	77.78(6)
O9-Mg2-O8	84.93(7)	O9-Mg2-O13	92.13(7)	O9-Mg2-O21	175.00(8)
O21-Mg2-O8	90.10(7)	O21-Mg2-O13	86.32(7)	O12-Mg3-O8	90.57(7)
O12-Mg3-O13	86.58(7)	O12-Mg3-O22	174.88(7)	O13-Mg3-O8	78.39(6)
O13-Mg3-O22	88.63(6)	O17-Mg3-O8	168.16(7)	O17-Mg3-O12	92.07(7)
O17-Mg3-O13	90.25(7)	O17-Mg3-O22	89.74(7)	O20-Mg3-O8	87.43(7)
O20-Mg3-O12	89.36(7)	O20-Mg3-O13	165.20(7)	O20-Mg3-O17	104.13(7)
O20-Mg3-O22	94.84(7)	O22-Mg3-O8	86.70(7)	O16-Mg4-O22	97.56(7)
O16-Mg4-O25	86.63(7)	O16-Mg4-O26	85.79(8)	O18-Mg4-O16	88.75(8)
O18-Mg4-O22	93.40(7)	O18-Mg4-O25	91.80(7)	O18-Mg4-O26	174.53(8)
O19-Mg4-O16	166.34(8)	O19-Mg4-O18	100.85(7)	O19-Mg4-O22	91.61(7)
O19-Mg4-O25	83.39(7)	O19-Mg4-O26	84.58(7)	O22-Mg4-O25	173.38(8)
O22-Mg4-O26	87.01(7)	Mg3-O13-Mg2	101.55(7)	Zn1-O21-Mg1	114.25(8)
Zn1-O21-Mg2	114.68(9)	Mg1-O21-Mg2	110.74(8)	Zn2-O22-Mg3	115.50(8)
Zn2-O22-Mg4	114.99(8)	Mg4-O22-Mg3	111.57(7)		

Table S3 Selected bond lengths (Å) and angles (deg) for **1-CHO**.

**Bond Lengths (Å)**					
Zn1-O6	1.9838(17)	Zn1-O8	1.9585(17)	Zn1-O9	1.9854(18)
Zn1-O11	1.9780(16)	Mg1-O1	2.0335(18)	Mg1-O3	2.0103(19)
Mg1-O7	2.0455(19)	Mg1-O11	2.0611(18)	Mg1-O11	2.0611(18)
Mg1-O12	2.1289(18)	Mg1-O13	2.1396(19)	Mg2-Mg2^{1}	3.2872(14)
Mg2-O2	1.9965(18)	Mg2-O4	2.0254(18)	Mg2-O5	2.0883(17)
Mg2-O5^{1}	2.1651(17)	Mg2-O10^{1}	2.0486(18)	Mg2-O11	2.1427(17)
**Bond Angles (deg)**					
O3-Mg1-O12	87.51(7)	O3-Mg1-O13	169.73(8)	O7-Mg1-O11	97.66(7)
O7-Mg1-O12	85.61(7)	O7-Mg1-O13	82.39(8)	O11-Mg1-O12	175.88(7)
O11-Mg1-O13	93.22(7)	O12-Mg1-O13	84.70(7)	O2-Mg2-Mg2^{1}	127.89(6)
O2-Mg2-O4	102.26(8)	O2-Mg2-O5^{1}	89.68(7)	O2-Mg2-O5	166.52(8)
O2-Mg2-O10^{1}	87.04(7)	O2-Mg2-O11	95.08(7)	O4-Mg2-Mg2^{1}	129.81(6)
O4-Mg2-O5^{1}	167.60(8)	O4-Mg2-O5	89.72(7)	O4-Mg2-O10^{1}	93.57(7)
O4-Mg2-O11	90.66(7)	O5-Mg2-Mg2^{1}	40.25(5)	O5^{1}-Mg2-Mg2^{1}	38.55(4)
O5-Mg2-O5^{1}	78.80(7)	O5-Mg2-O11	90.87(7)	O10^{1}-Mg2-	87.71(6)
O10^{1}-Mg2-O5	86.06(7)	O10^{1}-Mg2-O5^{1}	90.32(7)	O10^{1}-Mg2-O11	174.76(7)
O11-Mg2-	87.21(5)	O11-Mg2-O5^{1}	84.91(6)	Mg2-O5-Mg2^{1}	101.20(7)
Zn1-O11-Mg1	115.87(8)	Zn1-O11-Mg2	112.10(8)	Mg1-O11-Mg2	109.54(7)

Table S4 Selected bond lengths (Å) and angles (deg) for **2**.

**Bond Lengths (Å)**					
Mg1-O1	2.141(2)	Mg1-O2	2.145(2)	Mg1-O3	2.001(3)
Mg1-O5	1.977(2)	Mg1-O7	2.150(3)	Mg1-O8	2.067(2)
Mg2-O2	2.1276(19)	Mg2-O2^{1}	2.1276(19)	Mg2-O4	2.0458(19)
Mg2-O4^{1}	2.046(2)	Mg2-O6	2.0628(17)	Mg2-O6^{1}	2.0629(17)
Mg3-O9	2.1433(17)	Mg3-O10	2.1422(16)	Mg3-O11	2.0320(16)
Mg3-O13	1.9924(16)	Mg3-O15	2.0474(17)	Mg3-O16	2.1928(16)
Mg4-O10^{2}	2.1101(14)	Mg4-O10	2.1101(14)	Mg4-O12	2.0388(15)
Mg4-O12^{2}	2.0388(15)	Mg4-O14^{2}	2.0370(14)	Mg4-O14	2.0369(14)
**Bond Angles (deg)**					
O1-Mg1-O2	61.63(7)	O1-Mg1-O7	85.74(9)	O2-Mg1-O7	86.91(8)
O3-Mg1-O1	93.29(10)	O3-Mg1-O2	94.24(9)	O3-Mg1-O7	177.93(9)
O3-Mg1-O8	89.65(10)	O5-Mg1-O1	163.96(9)	O5-Mg1-O2	103.97(8)
O5-Mg1-O3	94.94(10)	O5-Mg1-O7	86.44(9)	O5-Mg1-O8	98.80(9)
O8-Mg1-O1	94.99(8)	O8-Mg1-O2	156.45(9)	O8-Mg1-O7	88.62(9)
O2-Mg2-O2^{1}	180.0	O4^{1}-Mg2-O2	90.11(8)	O4-Mg2-O2	89.88(8)
O4-Mg2-O2^{1}	90.12(8)	O4^{1}-Mg2-O2^{1}	89.89(8)	O4-Mg2-O4^{1}	180.0
O4^{1}-Mg2-O6^{1}	93.24(8)	O4^{1}-Mg2-O6	86.76(8)	O4-Mg2-O6	93.24(8)
O4-Mg2-O6^{1}	86.76(8)	O6-Mg2-O2^{1}	90.45(7)	O6-Mg2-O2	89.56(7)

$O6^1$-Mg2-O2	90.45(7)	$O6^1$-Mg2-$O2^1$	89.55(7)	O6-Mg2-$O6^1$	180.00(13)
O11-Mg3-O15	91.68(7)	O11-Mg3-O16	174.66(7)	O13-Mg3-O9	160.75(7)
O13-Mg3-O10	102.49(6)	O13-Mg3-O11	100.65(7)	O13-Mg3-O15	96.68(7)
O13-Mg3-O16	84.16(6)	O15-Mg3-O9	97.65(7)	O15-Mg3-O10	158.40(7)
O15-Mg3-O16	85.41(7)	O10-Mg4-$O10^2$	180.0	$O12^2$-Mg4-	90.30(6)
O12-Mg4-$O10^2$	89.70(6)	O12-Mg4-O10	90.30(6)	$O12^2$-Mg4-O10	89.70(6)
$O12^2$-Mg4-O12	180.0	O14-Mg4-O10	89.70(6)	O14-Mg4-$O10^2$	90.30(6)
$O14^2$-Mg4-O10	90.30(6)	$O14^2$-Mg4-	89.70(6)	O14-Mg4-$O12^2$	88.17(6)
$O14^2$-Mg4-O12	88.17(6)	$O14^2$-Mg4-	91.83(6)	O14-Mg4-O12	91.83(6)
O14-Mg4-$O14^2$	180.00(8)	Mg2-O2-Mg1	110.47(9)	Mg4-O10-Mg3	108.98(7)

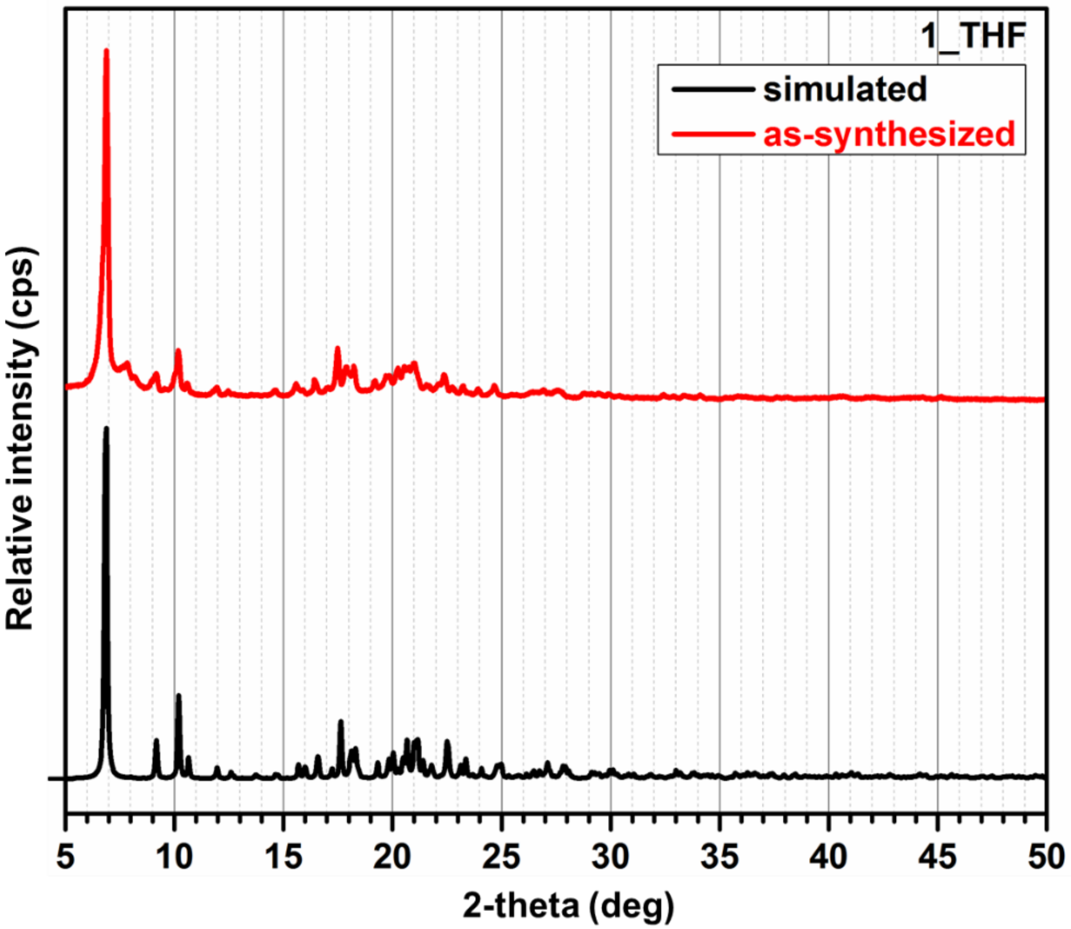

Figure S11. Powder X-ray diffraction pattern for **1-THF** compared with the simulated PXRD pattern obtained from single crystal X-ray diffraction analysis.

.

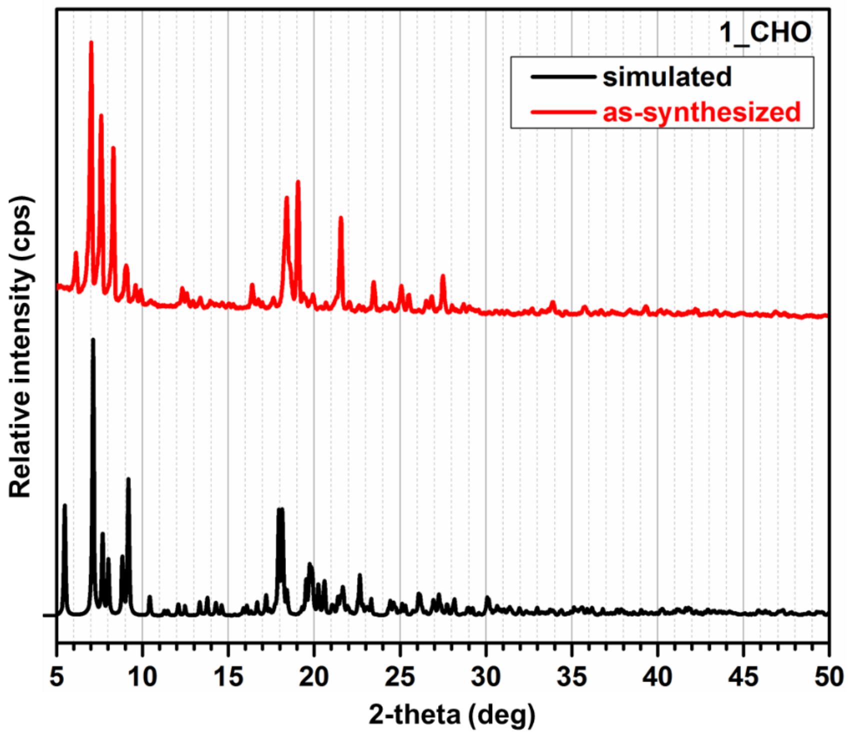

Figure S12. Powder X-ray diffraction pattern for **1-CHO** compared with the simulated PXRD pattern obtained from single crystal X-ray diffraction analysis.

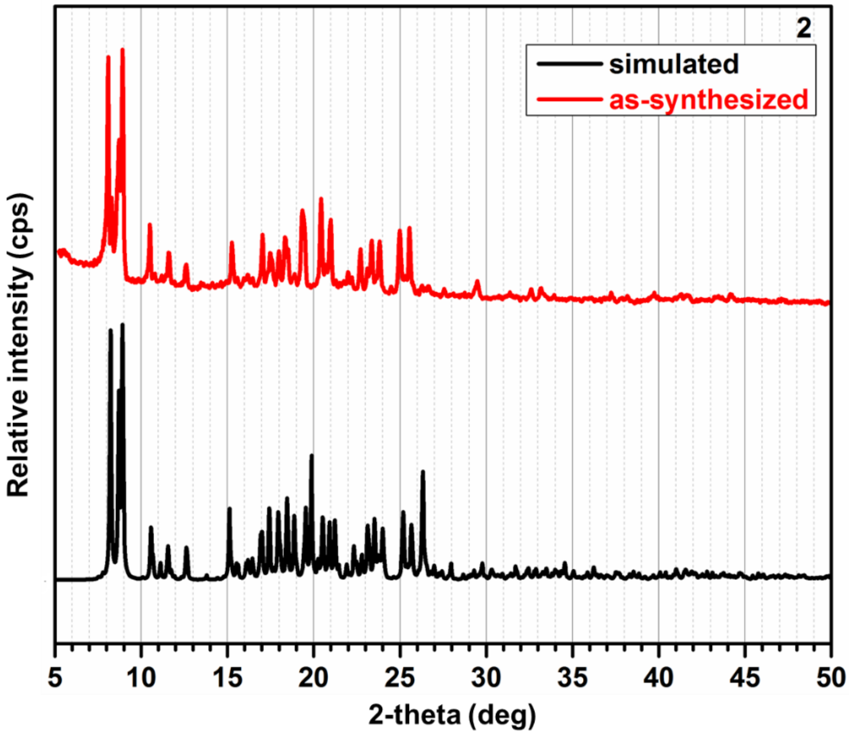

Figure S13. Powder X-ray diffraction pattern for **2** compared with the simulated PXRD pattern obtained from single crystal X-ray diffraction analysis.

Kinetics study for ROCOP

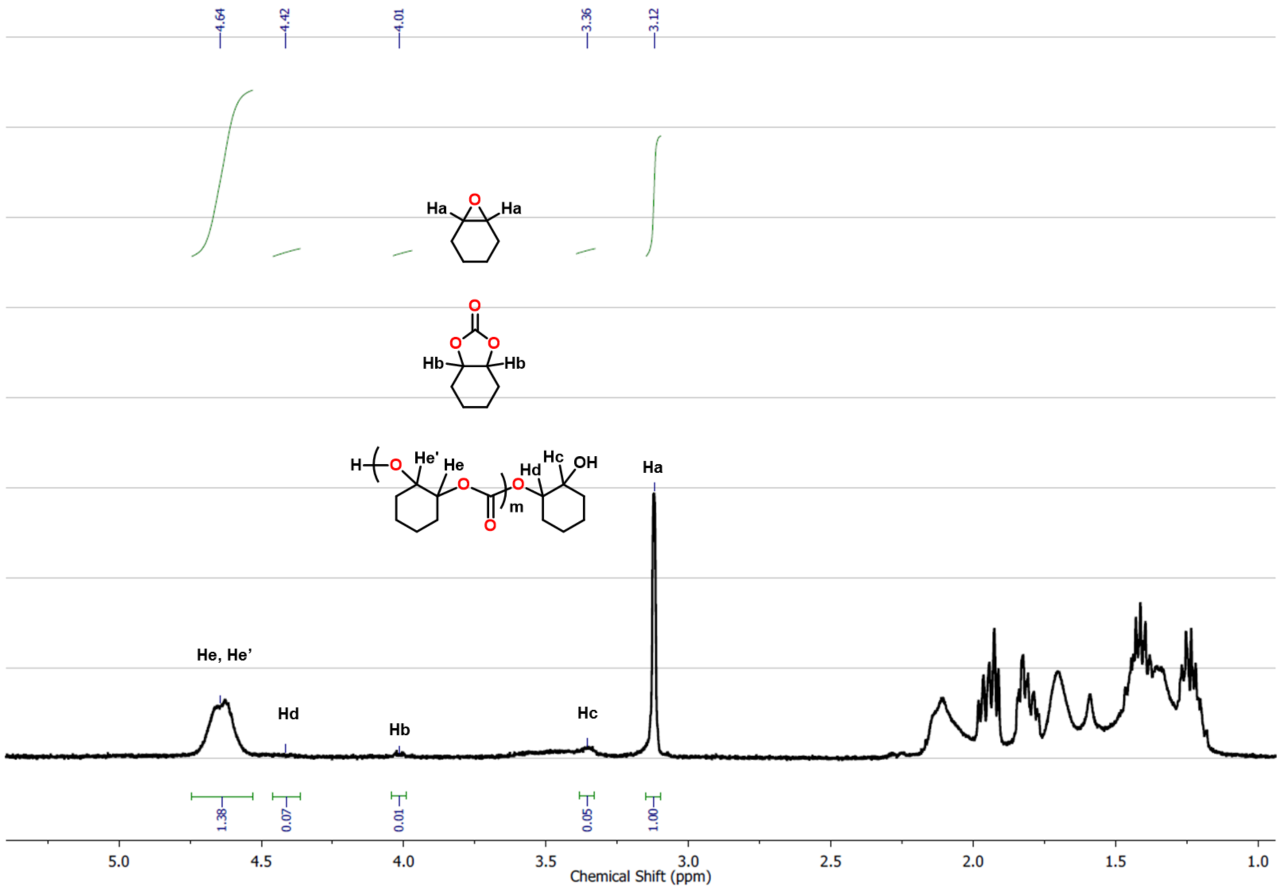

Figure S14. Typical ^{1}H NMR spectrum (in $CDCl_3$) used for determination of conversion and selectivity in ROCOP of CHO and CO_2 (Table 1, entry 3). The corresponding assignments are given in the inset molecular structures.

Calculation of the % conversion and selectivity in the ROCOP reaction

The characteristic peaks of α-protons (H_a and H_b) of cyclohexene oxide (reactant) and the corresponding cyclic carbonate (side-product in the ROCOP reaction) appears at δ 3.12, 4.01 ppm (trans-cyclic carbonate) and 4.68 ppm (cis-cyclic carbonate), respectively. Whereas, the characteristic peak of the polycarbonate appears at δ 4.64 ppm (H_e, $H_{e'}$). The peak at δ 3.36 ppm is due to any homopolymer formed in the reaction.

Total amount of compounds = Unreacted cyclohexene oxide + copolymer + homopolymer + cyclic carbonate) = 1+ 1.38 + 0.05 + 0.01 = 2.44

Percentage of the unreacted cyclohexene oxide: (100/2.44)% = 41%

Therefore percent conversion of cyclohexene oxide = (100-41)% = 59%.

Polymer selectivity = ((1.38 + 0.05)/(2.44 - 1)*100) = 99.03 %

Percent CO_2 insertion = ((1.38 + 0.01)/(2.44 - 1)*100) = 96.52 %

Table S5. ROCOP of CHO and CO_2 initiated by **1-THF**[a].

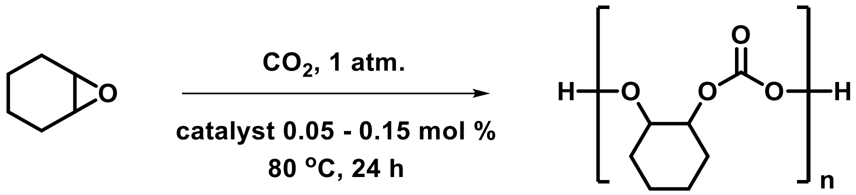

Entry	Catalyst loading ([CHO]/[cat.])	Conversion[b] (%)	CO_2[c]/Polymer[d] (%)	TON[e]	TOF[f] (h^{-1})	Yield of polymer (g)	Mn[Đ][g] ($kgmol^{-1}$)	Number of chains per Mg[h]
1	2000	37	96/>99	740	30.83	1.05	4.07[7.45]	1.30
2	1333	48	98/>99	640	26.66	1.36	4.20[11.6]	1.10
3	1000	59	97/>99	580	24.16	1.70	4.46[12.1]	0.96
4	666	70	97/>99	466	19.41	2.00	4.96[15.0]	0.81

[a]Reactions were run using different catalyst concentrations (0.05- 0.15 mol%) in 20 mmol of CHO (8 M in diethylcarbonate), 80 °C, 1 bar pressure of CO_2. [b]Expressed as percentage CHO conversion, determined from the ^{1}H NMR spectroscopy (Figure S14). [c]Expressed as a percentage of CO_2 uptake versus the theoretical maximum (100%), determined from the ^{1}H NMR spectroscopy by comparing normalized integrals for carbonate (4.64 ppm) and ether (3.45 ppm) resonances in the polymer backbone (Figure S14). [d] Expressed as a percentage of polymer formation versus the theoretical maximum (100%), determined from the ^{1}H NMR spectroscopy by comparing normalized integrals for polymer (4.65 ppm), cis-cyclic carbonate (4.68 ppm) and trans-cyclic carbonate (δ 4.01 ppm) (Figure S14). [e]TON = $mol_{CHO\ converted}$ x $(mol_{cat})^{-1}$. [f]TOF = TON per hour. [g]Determined by GPC analysis, in DCM, calibrated with narrow-Mn polystyrene standards, dispersity values in parentheses. [h]Number of polymer chains calculated from Yield/(Mn × Mg center).

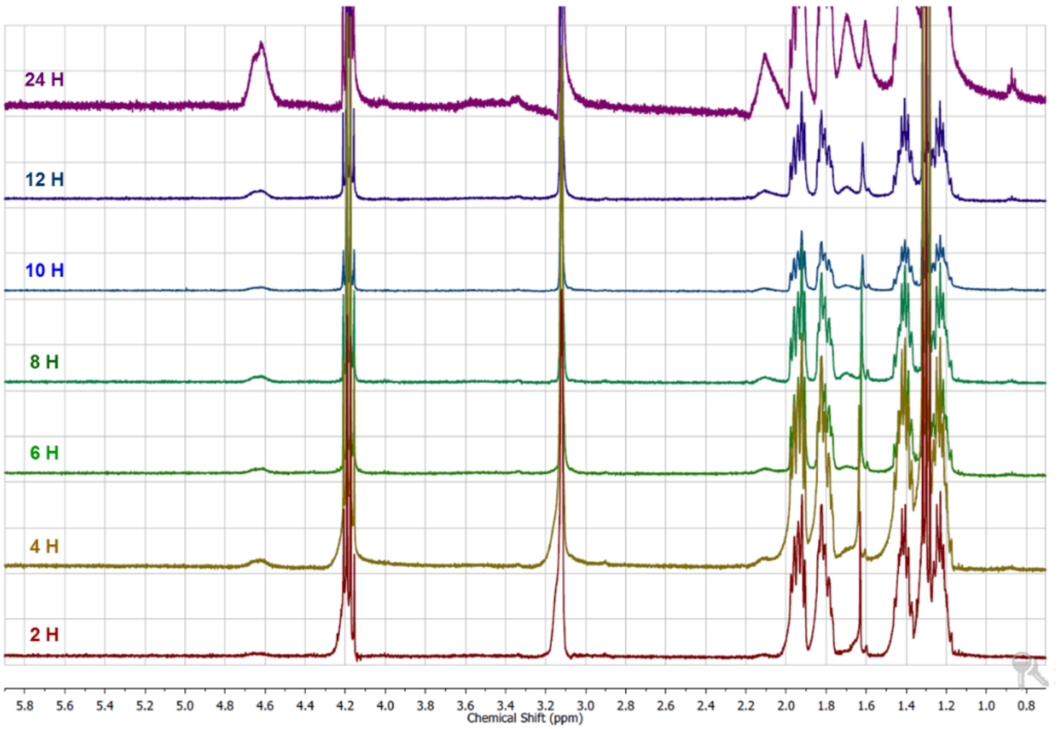

Figure S15. Monitoring of the reaction progress using ^{1}H NMR spectroscopy (in $CDCl_3$). Reaction conditions: 20 mmol CHO (8M solution in diethyl carbonate (DEC)), 1:2000 catalyst:CHO, 80 °C at 1 bar CO_2 pressure. The strong peak at 4.1-4.2 ppm corresponds to the protons of the solvent diethyl carbonate.

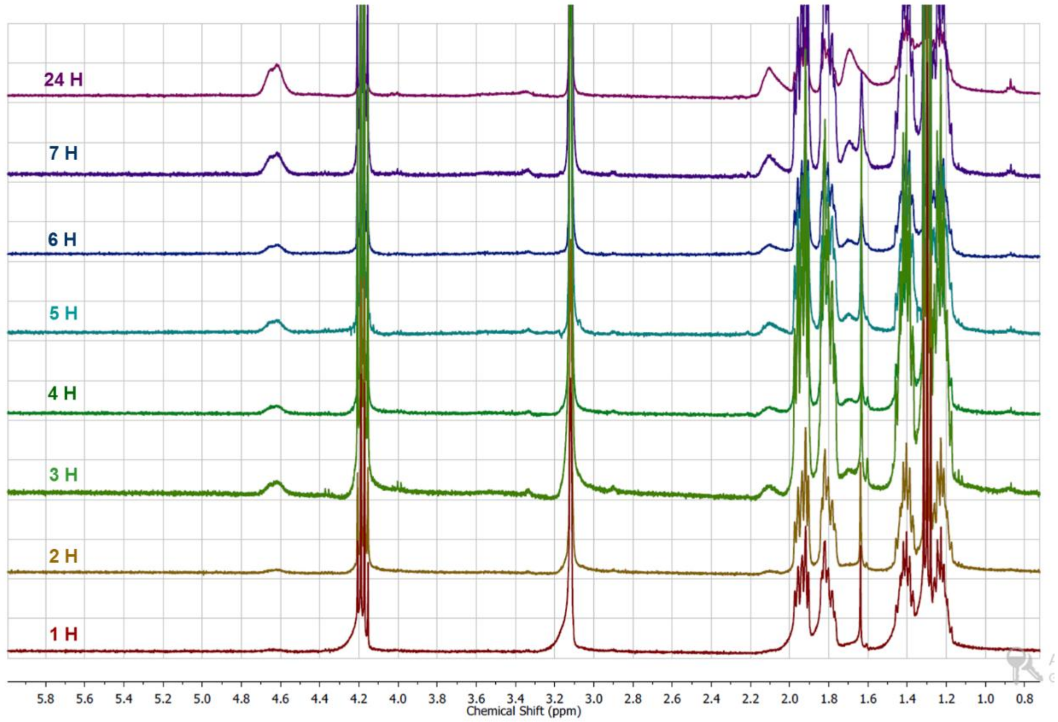

Figure S16. Monitoring of the reaction progress using ^{1}H NMR spectroscopy (in $CDCl_3$). Reaction conditions: 20 mmol CHO (8M solution in diethyl carbonate (DEC)), 1:1333 catalyst:CHO, 80 °C at 1 bar CO_2 pressure. The strong peak at 4.1-4.2 ppm corresponds to the protons of the solvent diethyl carbonate.

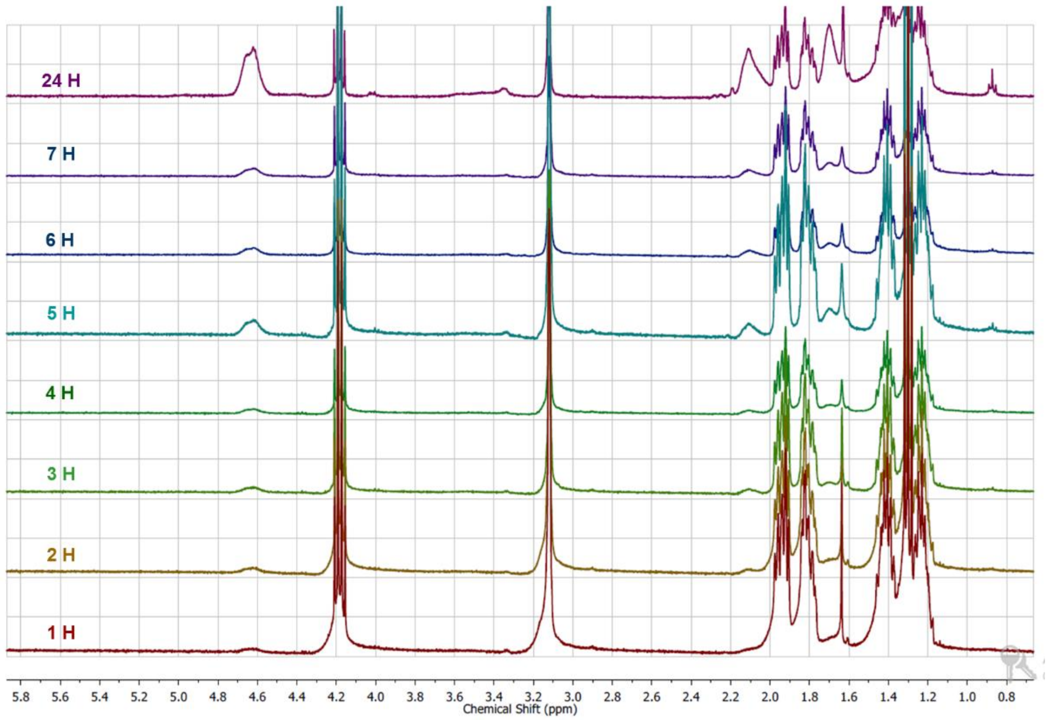

Figure S17. Monitoring of the reaction progress using ^{1}H NMR spectroscopy (in $CDCl_3$). Reaction conditions: 20 mmol CHO (8M solution in diethyl carbonate (DEC)), 1:1000 catalyst:CHO, 80 °C at 1 bar CO_2 pressure. The strong peak at 4.1-4.2 ppm corresponds to the protons of the solvent diethyl carbonate.

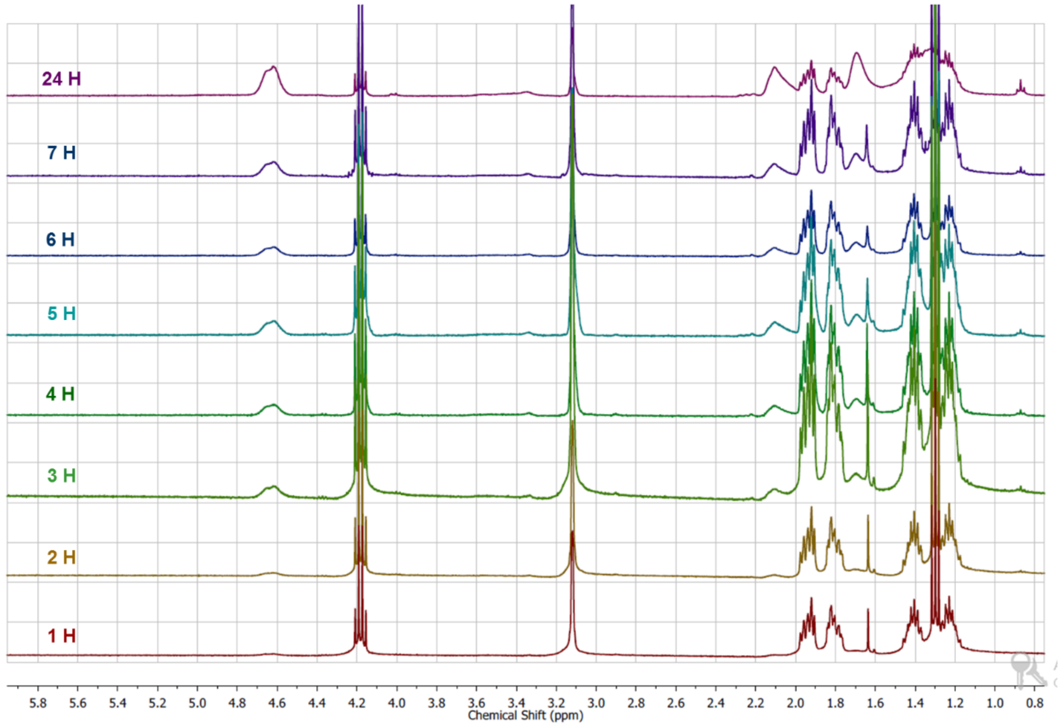

Figure S18. Monitoring of the reaction progress using ^{1}H NMR spectroscopy (in $CDCl_3$). Reaction conditions: 20 mmol CHO (8M solution in diethyl carbonate (DEC)), 1:666 catalyst:CHO, 80 °C at 1 bar CO_2 pressure. The strong peak at 4.1-4.2 ppm corresponds to the protons of the solvent diethyl carbonate.

Table S6. Kinetic studies of CO_2/CHO copolymerization catalyzed by **1-THF** at various monomer-to-catalyst Ratio (666-2000)[a]

Entry	Catalyst loading ([CHO]/[cat.])	Time (hrs)	Conversion[b] (%)	$\ln([CHO]_o/[CHO]_t$
1	2000	2	0.2	0.002002
2	2000	4	1.96	0.019795
3	2000	6	4.76	0.04877
4	2000	8	7.4	0.076881
5	2000	10	10.71	0.113281
6	2000	12	14.66	0.158527
7	2000	24	37.88	0.476102
8	1333	1	0.15	0.001501
9	1333	2	1.69	0.017044
10	1333	3	3.61	0.036768
11	1333	4	5.76	0.059325
12	1333	5	8.54	0.089268
13	1333	6	10.9	0.115411
14	1333	7	13.28	0.142486
15	1333	24	48.79	0.669235
16	1000	1	0.99	0.009949
17	1000	2	1.96	0.019795
18	1000	3	5.66	0.058265
19	1000	4	8.25	0.086103
20	1000	5	12.28	0.13102
21	1000	6	15.25	0.165464
22	1000	7	17.35	0.190555
23	1000	24	59.15	0.871078
24	666	1	3.54	0.036042
25	666	2	6.54	0.067637
26	666	3	11.09	0.117546
27	666	4	15.66	0.170314
28	666	5	20.87	0.234078
29	666	6	25.24	0.290887
30	666	7	28.47	0.335053
31	666	24	70.14	1.20865

[a]Reactions were run using different catalyst concentrations (0.05- 0.15 mol%) in 20 mmol of CHO (8 M in diethylcarbonate), 80 °C, 1 bar pressure of CO_2. [b]Expressed as percentage CHO conversion, determined from the ^{1}H NMR spectroscopy (Figure S14).

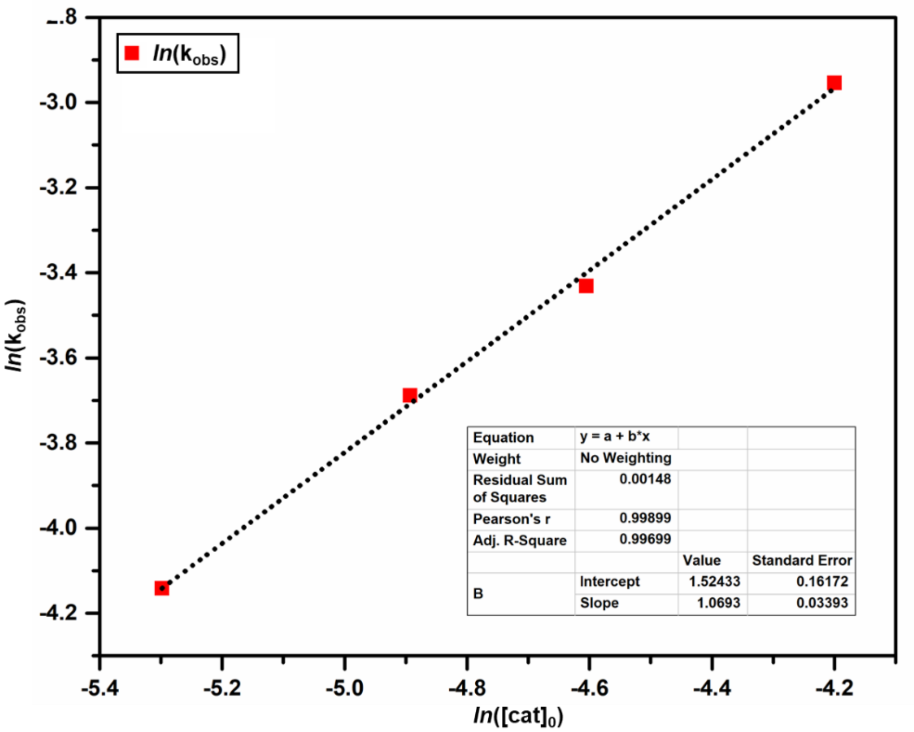

Figure S19. A linear plot of $ln k_{obs}$ versus $ln[cat]_0$ showing a first-order dependence on the catalyst concentration.

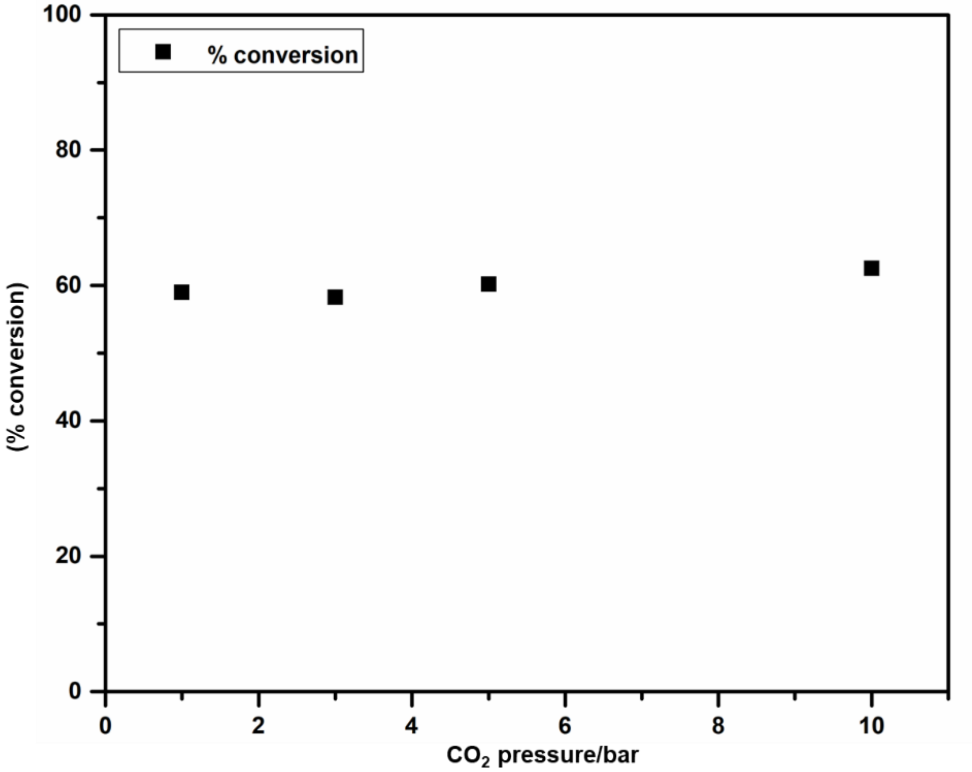

Figure S20. Conversion of CHO vs CO_2 pressure in the presence of 0.1 mol % catalyst at 80 °C for 24 h reaction.

References:

S1 Nilsson, M. The DOSY Toolbox: A new tool for processing PFG NMR diffusion data. *J. Magn. Res*. **2**, 296-302 (2009).

S2 Neufeld, R. & Stalke, D. Accurate molecular weight determination of small molecules via DOSY-NMR by using external calibration curves with normalized diffusion coefficients. *Chem. Sci*. **6,** 3354–3364 (2015).

S3 Bachmann, S., Neufeld, R., Dzemski, M. & Stalke, D. New External Calibration Curves (ECCs) for the Estimation of Molecular Weights in Various Common NMR Solvents. *Chem. Eur. J*. **22**, 8462–8465 (2016).

S4 Kreyenschmidt, A. K., Bachmann, S., Niklas, T. & Stalke, D. Molecular Weight Estimation of Molecules Incorporating Heavier Elements from van-der-Waals Corrected ECC-DOSY. *ChemistrySelect* **2**, 6957–6960 (2017).

S5 CrysAlisPro, Data Collection and Processing Software for Agilent X-ray Diffractometers, ver. 1.171.35.21b, Agilent Technologies, 2012.

S6 Sheldrick, G. M. A short history of SHELX. *Acta Crystallogr., Sect. A: Found. Crystallogr*. **A64**, 112–122 (2008).
